# Sub-diffractional infrared absorption of two-dimensional water

**DOI:** 10.1038/s41467-026-72629-9

**Published:** 2026-05-14

**Authors:** Marcos V. Surmani Martins, Hiran Jyothilal, Maximilian R. Becker, Ravalika Sajja, Ashok Keerthi, Roland R. Netz, Gianfelice Cinque, Boya Radha

**Affiliations:** 1 Department of Physics & Astronomy, The University of Manchester, Manchester, UK; 2 National Graphene Institute, The University of Manchester, Manchester, UK; 3 Fachbereich Physik, Freie Universität Berlin, Berlin, Germany; 4 Department of Chemistry, The University of Manchester, Manchester, UK; 5 Photon Science Institute, The University of Manchester, Manchester, UK; 6 Diamond Light Source Ltd., Chilton-Didcot, Oxfordshire, UK; 7 Department of Engineering Science, The University of Oxford, Oxford, UK

**Keywords:** Nanofluidics, Fluidics, Infrared spectroscopy

## Abstract

Several theoretical frameworks predict that water’s vibrational dynamics and dielectric properties undergo dramatic changes when confined to a monolayer. However, experimental validation has been limited, often relying on extrapolations from bulk water or multilayered systems at solid-liquid interfaces. Here, we overcome these challenges by using Ångström-scale slit-like capillaries formed within van der Waals heterostructures, enabling infra-red (IR) signal enhancement necessary to probe the vibrational signature of two-dimensional (2D) water using synchrotron IR micro spectroscopy. This enhancement arises from the high reflectivity of atomically flat graphite combined with the resonating waveguide modes accommodated by hexagonal boron nitride (hBN). For the extreme monolayer state, we show that the measured changes in water’s intramolecular vibrational modes reflects a significant frustration of the average hydrogen-bonding network. Furthermore, supported by DFT-MD, our data reveal the average structure of monolayer water and its evolution towards the bulk-state based on the population of the manyfold water clusters combined. Evidence of increased density, despite such disrupted hydrogen-bonding network, suggests the emergence of an unusually discontinuous phase. Our findings provide the first direct experimental evidence of water’s vibrational signatures and hydrogen-bonding network in 2D, offering new insights into the fundamental properties of confined water.

## Abstract (web summary)

The authors present direct observations of the vibrational spectra and structure of monolayer confined water. A frustrated hydrogen bonding network is revealed, offering insights into its long-elusive properties.

## Introduction

Water exhibits strong deviations in its properties when confined to ultra-small dimensions within nanochannels and at interfaces. Besides the extreme conditions imposed by confinement and interfacial forces, water’s unique electronic and nuclear structure contributes to a set of unusual properties that make it exceptional against most molecular systems^1,2^. The structure shaped by its hydrogen-bonding (HB) network at lower dimensions, defines water’s energy landscape and gives rise to the unusual physical-chemical properties^3^, such as ultrafast flow^4–6^ and a highly anisotropic dielectric function^7–9^. An appealing strategy to fully uncover the origin of such anomalies is to study the effects of dimensionality on water’s HB, so that the large degrees of freedom are suppressed. However, despite all the efforts and often use of terms such as “two-dimensional” and “Ångström-confinement”, direct experimental evidence on how the structure evolves in a truly isolated single-layer of water is still lacking, and still relies on theoretical predictions and extrapolations^7,10,11^.

For decades, vibrational spectroscopy has been an effective tool for probing water’s structure and HB environment. In particular, the water molecule stretching modes offer a detailed picture of its HB network, revealing distinct changes compared to bulk water as it is scaled down to smaller number of molecules or at interfaces^12–16^. Nonetheless, the limited cross-section in the actual monolayer regime is a major challenge, preventing these subtle structural features from being experimentally resolved, even by state-of-the-art spectroscopy techniques. In fact, sum-frequency generation (SFG) spectroscopy recently emerged as a promising approach due to its inherent interface sensitivity^17–20^. In the 2D confinement regime, the opposing confinement interfaces results in either no SFG signal for symmetric slits formed by the same material, or a rather complex difference spectrum for asymmetric ones^19^. Thus, despite the limited cross-section, linear vibrational methods such as infra-red (IR) absorption spectroscopy still forms an advantageous route as it directly measures the molecular vibrational modes from a straightforward one-photon process.

Developing a suitable host structure to study the properties of pristine water single layers to unravel the elusive properties of HB network has long been a challenge. Crystalline laminate materials such as graphene oxide^21^, clays, gypsum and other crystalline systems constitute potential candidates and were extensively used to fulfil such role in X-Ray and neutron crystallography as well as vibrational spectroscopy^12,22–27^. However, strong correlations between water and these hydrophilic environments outweighs the water-water correlations framing the bare HBs, which thus remained obscure. The discovery of 2D materials and their portfolio of features such as being atomically flat and only mildly hydrophilic^28^ holds good promises in this respect.

As two of the primordial in the vast suite of 2D materials, graphene and hexagonal boron nitride (hBN) still attract great interest due to their contrasting electronic and optical properties despite sharing structural similarities in real space. In the IR range, while graphite and graphene display a rather semi-metallic behaviour with a negative electric permittivity thus high reflectivity, hBN is a natural hyperbolic material capable of hosting long-range phonon-polaritons confining the IR light within the Reststrahlen bands, and strong resonating waveguide modes across the whole THz-IR range^29–34^.

With the recent advances in nanofabrication, manipulating 2D materials with single-layer precision triggered the ambitious goal to combine them into heterostructures to exploit their properties and develop confined nanofluidic platforms for studying ionic and molecular species in the sub-nanometre scale^35–40^. In this aspect, using graphene and hBN as building blocks for assembling such confining environments with highly accurate channel geometry down to atomic scales has become attractive. These Ångström-scale channels are an excellent platform for studying properties of static and flowing ultra-confined matter such as water, gases, and ions at the dimensions of a true single layer. Under such conditions, intriguing properties of water were revealed, including fast water flows^6,41^ and anomalously anisotropic dielectric constant in a static regime^9,7^. In comparison with the strongly correlated host lattices mentioned above, and because of their moderately hydrophilic surfaces, intermolecular interactions between guest water molecules in these structures are not screened away by the effects of the host environment. Such feature is crucial to disentangle the actual confinement effects from those emerging at interfaces.

Here, we probe the vibrational signature, structure, and interface interactions of ultra-confined phases of water down to a single layer (2D). By using atomically flat Ångström-capillaries with IR amplifying properties, we reveal the formation of independent single water layers and probe their vibrational signatures and HB structures using high brightness synchrotron-IR micro-spectroscopy in reflectance mode. We used a particular configuration of van der Waals heterostructure comprising atomically flat capillaries disposed as “empty space” between hBN and thin graphite crystals. Such slit-shaped channels are fabricated with atomic precision and with thicknesses down to an equivalent graphene monolayer, able to accommodate a single water layer. The combined properties of graphite and hBN as an atomically flat reflective layer and an efficient waveguide, respectively, secured the amplifying feature in the infra-red range, revealing water’s vibrational absorption bands with superior signal-to-noise ratio spectra. We further present the water structural evolution across several channel thicknesses and demonstrate a sharp transition, where a superposition of interfacial and bulk effects starts dominating above ~1 nm (3 graphene-layers thick channels). We combine the direct vibrational measurements of the water -OH stretching band with density functional theory (DFT)-based molecular dynamics simulations to unveil the hydrogen bonding structure, interface *versus* confinement effects, and spectral properties of a true monolayer water. Our experimental results show that the correlated modes are strongly supressed at the monolayer regime thus revealing a rather disordered hydrogen-bonding structure, diverging from what is known for common interfaces.

## Results

### Squashing water into a single layer

**Fig. 1:**
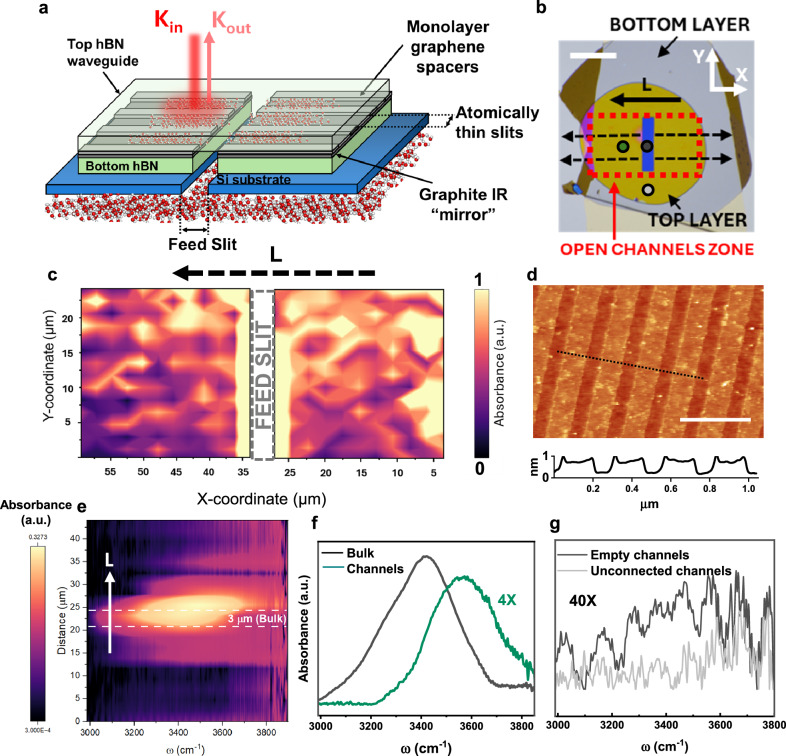
Real space IR imaging of monolayer water confined in Ångström-scale capillaries. **a** Schematics of van der Waals materials forming the heterostructure comprising the Ångström-scale capillaries. Monolayer graphene spacers are distributed across a thin graphite layer topped with hBN crystal. A hBN bottom layer is used to secure the adhesion of the device onto the substrate. A 3 × 25 μm^2^ slit across the Si substrate is used to feed the capillaries and give access to the bulk water for direct comparison. K_in_ and K_out_ denote the incoming and outgoing synchrotron IR microbeam, respectively. **b** Optical image of a monolayer device displaying the graphite and hBN bottom and top layer, respectively. The coordinate frame with respect to the stage is represented by x and y. Scale bar is 20 μm. The black arrow defines the scan direction L for the spectral map presented in (**c**), measured across the red dashed box. **c** Hyperspectral intensity distribution of the 3000–3800 cm^−1^ range (water’s stretching mode), across the red dashed box displayed in (**b**) confirming the pattern of the capillaries. **d** AFM height distribution and profile along the spacers forming the capillaries. Scale bar is 500 nm. **e** One-dimensional spectral distribution across the feed slit and along the direction L indicated in (**b**). **f**, **g** Sample spectra along the stretching mode frequency range corresponding to the locations marked in (**b**) for the bulk water across the feed slit and water confined along the monolayer capillaries are shown in (**f**). The control spectra, measured from the capillaries before water fills the device and from the unconnected capillaries for filled device, is presented in (**g**).

A schematic of the device structure and far-field IR micro-spectroscopy experiment in reflectance mode is displayed in Fig. 1a. The measurements were performed using synchrotron IR microprobe in scanning microscopy mode by slits defining the detected area onto the sample surface. The detailed fabrication protocol of the nanochannels is available elsewhere^42^ and also described in Methods. Briefly, the devices were directly fabricated on bare Si as an ideal substrate in the mid-IR range. We used a thin hBN layer supporting the devices onto the substrate to enhance its adhesion thus securing its air/water-tight feature that allows water to only flow across the capillaries. Water was directly fed into the capillaries from a 3 × 25 μm^2^ slit etched through the Si substrate and later dry-etched across the bottom layer before the top is transferred.

The large refractive index of graphite in the mid-IR range^33,34^ was the primary property considered when designing the base of the capillaries in the heterostructures. Graphene spacers separate the hBN wave-guiding top layer from the highly reflective graphite bottom layer. The number of graphene layers forming the spacers defines the total height of the 2D capillaries (see AFM topography in Fig. 1d) where a monolayer graphene is set as the limit to accommodate a single layer of water. The chip was mounted onto a custom-built air/water-tight cell, which was directly loaded onto the IR microscope’s sample stage (Fig. S2). For background, reference, and calibration, we intentionally maintained a large population of capillaries unconnected to the feed slit, so that these remain empty throughout the scans (Fig. 1b, grey circle) and the relative true absorption from the confined water only could be accessed. To eliminate any effects from fluctuations in the measurement conditions over time, background spectra on the empty channels were collected after every ten points of the hyperspectral dataset. Besides the inherent empty channel reference, such device configuration also provides a direct reference with the water’s bulk signal as the top-layer participates as a window across the feed slit. A bulk-to-confined comparison can, therefore, be directly established already ruling out effects from the top hBN layer thickness and instrumental broadening.

Figure 1f displays a representative spectrum in the -OH stretching regime from the water-filled channels in a monolayer device compared with the bulk water signal measured on the feed slit zone of the same device, through the top hBN layer. The spectral positions are marked in the optical image in Fig. 1b. In Fig. 1g, we confirm the complete absence of the -OH stretching mode band in both empty-channel states: (i) unconnected channels within a water-filled device (grey spot in Fig. 1b) and (ii) an empty channel in the dry state, before water filling and outside the water cell. The waveguide feature of hBN turns evident across empty channels. Under dry conditions, Fabry-Perot spectral fringes^43^, which underpins the wave-guiding feature of hBN arise above the baseline as also shown in Fig. 1g. The hyperspectral image of the monolayer device, shown in Fig. 1c, reveals the intensity distribution in real space, which follows the pattern of the water-filled channels within the red box marked in Fig. 1b. The dataset not only verifies the presence of water across the 2D capillaries even at the single layer regime but also validates the combined functionality of the heterostructure as an efficient signal enhancer in the mid-IR range.

Although the wave-guiding properties of hBN are sharper across the IR spectrum, it also extends into visible and near UV range. In fact, multiple works recently explored it as means to improve spectroscopic detection in systems with small absorption cross-section^44–46^. Thus, to further validate the presence of water confined in the channels, we further probed the stretching modes with a Raman microscope. The results are presented in the supporting information, Fig. S11. Besides the sensitivity limitation of the Raman scattering process versus a direct IR absorbance for spectral purposes, a few other points must be considered for our devices towards conventional Raman setups. One is regarding the heating caused by the laser, where temperature fluctuations could prevent us to probe the actual state of the confined water phase at the same conditions of the bulk used as reference. Another limitation refers to the Raman response of graphite. The overlapping D” band of graphite at 3250 cm^−1^ leads to a poor signal-to-noise ratio of the -OH stretching mode in thinner channels and the amplitude only becomes reliable for larger water populations in thick capillaries. Figure S11 reveals a line scan extending across the feed slit and the channels region measured with a 325 nm laser without the graphite layer. Despite its good agreement with the IR response for such multilayer devices, the shifts are much larger and could rather be related to actual water vapour formed upon the laser heating as the power density needed for a Raman spectrum can be order of magnitude (10–100 mW) higher than IR (total synchrotron IR power at the sample ~1 mW under B22 microscope). In such case, disentangling heating effects from the actual confinement effects becomes challenging. These results, however, validate both the presence of water in the capillaries as well as the optical robustness of the devices. Any concerns about the substrate’s transparency can be ruled out, as it is completely opaque at these wavelengths.

While the Raman scattering process (a two-photon process) depends on molecular polarizability, which is determined by charge redistribution upon excitation, the IR absorption (a one-photon process) is governed by the molecular dipole moment direct excitation. Due to the large dipole moment of water and its clusters, the intense IR response is as highly sensitive to their orientation as it is to the hydrogen-bonding structure, including interactions within water dipole condensates and with the surrounding confinement environment. Mid-IR spectroscopy allows access to a broader range of vibrational transitions with ${{\hslash }}{\rm{\omega }}\approx$ 10^2^ to 10^4 ^cm^−1^, which are strongly influenced by both the molecular structure and the surrounding forces. By analysing these normal modes, one can in principle, infer an average molecular structure within the confined space. Synchrotron IR microbeam proved ideal for diffraction-limited resolution and broadband FTIR spectroscopy without radiation damage, thus minimising the challenges caused by the limited amount of water molecules and/or independent layers in our devices, as well as avoiding any experimental fluctuations induced by the measurement acquisition, e.g., no local thermal effect.

The use of hBN as an IR-responsive photonic crystal has been widely studied and its resonating properties unravelled both experimentally and theoretically over the past years^29–31,47^. In fact, a similar goal motivated Bylinkin et al. to exploit these properties and push the detection limit of nanometre thin layers of organic molecules using near field approaches^48^. In their system, however, they focused on the hyperbolic response of hBN, where the signal was probed via momentum transfer from the surface phonon-polaritons propagating through hBN, to the molecular vibrations activated underneath it. Here, we purely rely on the resonating properties and discard any contribution from phonon polaritons of hBN, as we are probing frequencies matching the water’s -OH stretching modes, which are considerably far from the hyperbolic regime. Our approach also evolves from pure IR absorption, which is linearly correlates with the molecular vibrations, rather than higher order combined scattering such as in near field tip-based approaches. In addition, the high sensitivity offered by synchrotron IR microbeam became essential for probing our devices’ channels and remarkably made possible to achieve reliable spectra even in the extreme case of a single water layer in far-field IR detection mode. In simplified terms, such amplifying capacity of these heterostructures acts by essentially boosting the vibrational absorption detection, thus opening a new avenue for further studies on other relevant species.

### Spectral signature of water under extreme 2D confinement

**Fig. 2:**
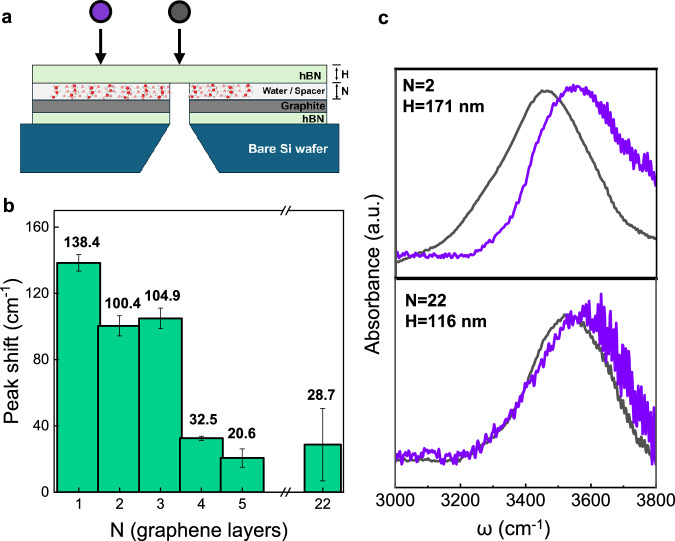
Experimental frequency shifts due to confinement effects. **a** Schematics marking the spectral locations of the water confined in the Ångström-scale capillaries in comparison with the bulk from the feed slit region of each device. N denotes the number of graphene layers of the capillary and H, the thickness of the top hBN layer. **b** Peak shift of the –OH stretching mode from several devices as a function of the capillary height in terms of N. The shifts are measured with respect to the bulk for each device thus ruling out any influence of the hBN top layer. The data are presented as the mean values ± the standard error of the mean (SEM) for the spectral sample. Mean values were obtained from multiple spectral measurements on each device, with the total number of spectra accommodated in both regions-of-interest (ROI), i.e., along the feed slit for measurements on the bulk and on the adjacent angstrom/nano-channels for confinement. In both ROIs, the number of pixels/spectra in the line scan was kept equal, ranging from 8 to 12 measurements for each. **c** Comparative spectra of the stretching modes for bilayer and 22-layer thin channel devices with their respective bulk signal.

To probe the vibrational features and HB arrangement across the confined water layers, we track the -OH stretching modes active in the 3000–4000 cm^−1^ (372–496 meV) range for channels with different thicknesses. As shown in the representative spectrum from the monolayer case in Fig. 1e, the peak blue-shifts when under confinement. Such effect is not exclusive to the extreme monolayer water case and persists as the channel thicknesses evolves towards the bulk-like regime. Data in Fig. 2b shows the magnitude of the peak shifts for several channel thicknesses given in number of graphene spacer layers (N). These values are obtained relative to the bulk measured at locations along each device’s feeding zone which is micro-hole covered with top hBN (see schematics in Fig. 2a). The resonating effects from the top hBN layer on the individual band positions are summarised in Fig. S4 by comparing the values for bulk water measured across the different hBN thicknesses (H) of a large population of devices with that of what is known for “bare liquid water” ( ~ 3400 cm^−1^, 421 meV). Here, such effects are ruled out as we focus on the shifts in terms of values relative to the bulk measured across the same top hBN layer for each device. This approach is not only practically convenient, by eliminating the need to distinguish individual reference band positions and minimising instrumental errors, it also provides the relevant insights into the energetics of the system towards lower dimensions.

In Fig. 2, large blue-shifts appear as we scale down to a single water layer, with peak positions moving towards higher frequencies suggesting significant changes in the HB network. This effect saturates as we increase the channel thickness and the HB network is recovered towards its bulk-like configuration, where only interfacial effects remain. Figure 2c reveals the stark contrast in the relative spectral features for a bilayer and a 22 layered (8 nm thick) capillary. Specifically, the magnitude of such blue-shifts in our data indicates that a rather disordered phase emerges as we scale down to the monolayer configuration, with fewer HBs per water molecules in average. Such confinement-to-bulk transition agrees well with a recent theoretical study on water confined between graphene layers probed via THz and IR absorption^49^. Here, with a step further into the sub-nanometre range, we experimentally validate these findings on confinement effects on the spectral response taking place at confinement thicknesses below 1.4 nm (N = 4). The steep increase in the blue-shifts for N < 4 values in Fig. 2b is evident and consistent with their findings despite the use of hBN as top layer in our channels.

Similar effects have also been measured for water confined in carbon nanotubes (CNTs)^50^ where the stretching mode was measured for confining diameters down to 1.4 nm. These measurements were obtained using a completely different technique, vibrational energy loss spectroscopy (vEELS), and further supported by DFT-MD simulations. In this one-dimensional analogue, the authors also found that bulk effects vanish for CNT with 1.4 nm in diameter and a disordered water phase emerges. These findings help refine our understanding of how water molecules behave in hydrophobic spaces with dimensions comparable to the hydrogen-bond (HB) length, a highly significant yet elusive concept with implications for many systems.

**Fig. 3:**
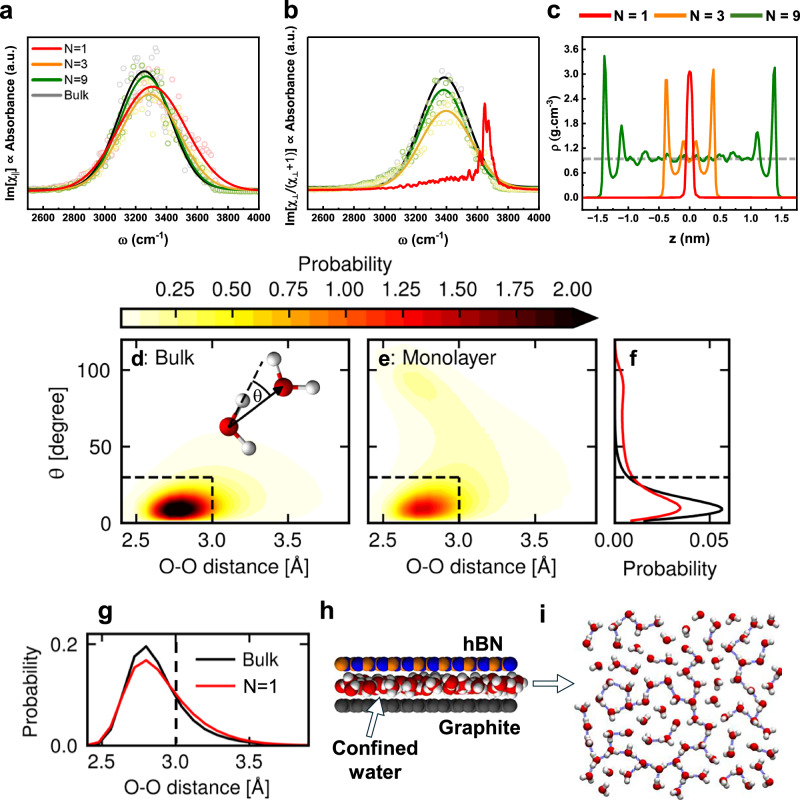
DFT-MD results for spectra and structure of water confined between graphene and hBN sheets. **a**, **b** Calculated in-plane (**a**) and out-of-plane (**b**) response of the –OH stretching bands for different confined water thicknesses based on N. The total absorption values are proportional to the imaginary terms of the in-plane response function (${\chi }_{\parallel }$) and out-of-plane response function [${\chi }_{\perp }$/ (${\chi }_{\perp }$ + 1)], where ${\chi }_{\parallel }$ and ${\chi }_{\perp }$ are the respective electric susceptibilities in each case. **c** Mass density profile along the normal (out-of-plane) direction z for various levels of confinement (N = 1, 3, and 9). The grey dashed line indicates the reference value that is known for the bulk. **d**–**g** Correlation maps calculated for bulk and monolayer confined water in hBN-graphite nanochannels. The maps depict the probability density for the overall hydrogen-bonding structure as a function of the angle formed between water molecules in donor-acceptor pairs (θ) and the distance between the respective oxygen atoms (O-O). The dashed lines correspond to a standard geometric criterion for hydrogen bonds, used to quantify the degree of hydrogen bonding. **h**, **i** DFT snapshots of the collective structure of monolayer confined water – *(h)* cross-sectional and *(i)* top view.

It is worth noting that, despite the high brightness of the synchrotron light used in our measurements and the enhancements promoted by the device’s structure discussed earlier, the area probed over a single water layer is yet fairly small if the density at the conditions of the measurements (room temperature and ambient pressure) is considered for either liquid or vapour phase. Therefore, we build the hypothesis that such intensities could also be justified if a relatively larger absorption cross-section is considered, where a rather packed water phase emerges despite such disordered structure. To better frame the distribution of water molecules under such conditions, we calculated the mass density profiles shown in Fig. 3c. As we scale down to the monolayer regime, a noticeable increase in density is suggested when compared with what is known for the bulk liquid water at room temperature. Such effect is in good agreement with the extensive study recently made by comparing both force-field (FF) and DFT-based MD simulations^49^. For channel thicknesses above 1.4 nm, bulk effects take place, and the densities associated with the layered hydration structure starts dominating.

Although the stretching mode is vastly explored due the robust set of information it provides, the bending mode of water has also caught attention in the recent literature^51^. Advances in simulations has shown that this mode can be a supporting beacon as powerful as the -OH stretching mode for probing both the HB network of water at interfaces and confinement as well as dissipative effects between water molecules^52,53^. In our case, as the bending bands fall within the frequency range of the hyperbolic response of hBN with strong fluctuations, it becomes less evident for the absorption cross-section measured on the mono- and bilayer water regimes. However, this band becomes evident for capillary thicknesses of a 3-layered device with ~1 nm thick capillaries as revealed in Fig. S6. These data are in excellent agreement with our calculations and further validates what has been proposed by an earlier theoretical study on the anisotropic susceptibility of water under 2D confinement. In the bending case, however, it opposes the energetic response of the stretching modes, and rather red-shifts are observed. While interfacial effects dominate at thicknesses larger than 1.4 nm, the magnitude of the shifts in our data line up with the theoretical predictions that 1 nm marks the threshold for geometrical confinement effects to start becoming effective. Despite our focus on the stretching frequencies, however, these results not only confirm and validate the authenticity of our data and calculations but also opens a new path to unravel the HB effects on the yet unexplored bending mode at lower dimensions.

In the following section, we further explore the origin of the spectral shifts (Fig. 2) and introduce a theoretical analysis to deepen our insights into the energetics and structure of these first-time observed monolayer water phases.

### The structure of two-dimensional water suggests an unusually disordered phase

The overall -OH stretching band is well known as a superposition of multiple normal modes with its frequency/position defined by the number of water molecules participating in the donor-acceptor configuration of the HB network^54^. For bulk water, the combined stretching band centred at ~3400 cm^−1^ builds as a superposition of different sub-modes^14–16^, each one related to a distinct HB environment. Although the exact positions of these sub-modes are still debated^54,55^, the position of the overall stretching band does depend on their relative intensity. For example, as HB weakens or the network is disrupted, for instance when water goes from liquid to vapour, a increased potential curvature leads to a shorter thus stiffer O-H bond^56^. A blue shift is then observed since high frequency sub-modes dominate as more energy is required to change water’s dipole moment. Such feature is observed in both water vapour and non-uniform condensates in hydrophobic surfaces^57^. The opposite, red-shifted peak, therefore, applies to strong HB water structures such as those in ices and hydrophilic interfaces^58^.

Our data in Figs. 1c, e, f and 2b, c are in good agreement with the spectra predicted from the simulations. Moreover, the off-normal incidence used in our experiment provides flexibility to explore the anisotropic components of the system combined. Contrasting electric susceptibilities between the in- and out-of-plane components has been extensively proposed and further probed experimentally for the near-2D regime^9,7,49^. In the limiting monolayer case presented here, bulk contributions can be fully discarded and only the in-plane HB network partially remains whereas in the out-of-plane directions water bears resemblance to its free state. As demonstrated by ref. ^50^ using an extensive DFT-MD study, such asymmetry imposes strong effects on the HB potential landscape. An increased curvature of the effective -OH bond potential in both directions is suggested to underlie such HB disruption and, therefore, blue shifts of the -OH stretching modes. The relation between the HB extent and the -OH bond length has been extensively studied as the latter defines the stiffness and, therefore, the extension of the dipole moment vibrational frequency of stretching modes. The calculated evolution of the -OH bond length along with the average number of HB per water molecules in our systems is presented in Fig. S7. In the monolayer regime, shorter thus stiffer-OH bonds dominate whereas lengths that are close to the bulk unfold towards larger channel thicknesses thus agreeing well with the experimental values presented in Fig. 2b. Moreover, as water deviates from the tetrahedral coordination stabilised in the bulk, we note that the number of HB per water molecule in such disordered single layer phase drops substantially with an average between 2 and 3 HBs.

The blue shifts observed in our measurements when going from bulk to monolayer water are also evidenced in the DFT spectra presented in Fig. 3a, b for both in- and out-of-plane directions. Such distinction is convenient as it reflects the response of the confined phases under high geometrical anisotropy, especially for the limiting monolayer case where the channel dimensions comparable with water’s van der Waals diameter only allows these two molecular orientations. Notably, a broad in-plane absorption spectrum is observed even in the monolayer case suggesting that an ample distribution of hydrogen bonds is formed parallel to the graphene/hBN plane. On the other hand, a sharp -OH stretch peak, characteristic of dangling -OH groups, stands out for the out-of-plane direction indicating that the population of molecules disposed perpendicular to the graphene/hBN planes are rather unconnected from the HB network. In our experiment, however, the off-normal IR incidence result in a superposition of both orientational signals, where the broader in-plane response dominates (see setup and optics in Fig. S2). Therefore, although the combined blue shifts are clear, we are unable to make a clear distinction of the sharp dangling -OH peak.

To unveil the structural moieties forming the monolayer water phase and its HB network, we perform a statistical analysis over the HB donor-acceptor (D-A) pairs, where we correlate the calculated HB angular distribution with their length (O-O distance). Here, the HB angle θ is defined by the orientation of the hydrogen atom relative to the O-O axis of the D-A pair as schematically depicted in the inset of Fig. 3d. The data is summarised in Fig. 3d–g framing the correlation map obtained for the monolayer phase in direct comparison with the bulk counterpart. In both cases, the hottest regions concentrate below 30 degrees angle and 3 Å in length, which are well known as the most stable water’s configuration at room temperature. However, for the monolayer regime, the histogram is evidently re-distributed, with an additional population emerging with much larger angles as indicated by the spread wing in the projected plots in Fig. 3f.

Such distortion is not surprising since the physical sub-nanometre constraint prevents the formation of the tetrahedral HB structure as it is pushed down into the planar configuration. In this scenario, the ideal HB angle is then suppressed for a considerable population of water molecules as the pairs stabilise rotated from their ideal angle, thus leading to a frustrated network with fewer HB per water molecule accommodated across un-bonded water sites. Such mosaic-like structure is presented in the snapshots in Fig. 3i. Interestingly, as this disrupted network comprises a large variety of sites having from none up to 3 HBs, a rather broad spectral response such as those observed in our measurements are expected since the -OH stretching frequencies vary slightly for each HB state.

The way how water clusters deviate from their ideal tetrahedral arrangement upon physical or environmental effects turns out as a rich structural descriptor, which has been vastly used over the recent years, especially upon phase transitions and interfaces^59–64^. Thus, to refine our understanding on how the structure evolves towards a true monolayer state, and whether the interfaces enclosing our capillaries exert any effects, we take a closer look into the ordering of the water phases at both interfacial and pure confinement regimes. We analyse such correlations in terms of the common tetrahedral order parameter popularised by Errington & Debenedetti^65^, which has been recently adapted by Wang et al*.*^66^ for the use with interfacial water. The parameter q takes into account the tetrahedral undercoordination given by different angles formed between neighbouring water molecules. In brief, q varies between 0 and 1 so that q = 1 reflects a perfectly tetrahedral coordination of water molecules, whereas q = 0 the total absence of order as expected for a gas phase. A full description of the calculation is presented in Methods.

Overall, we find stark differences in the hydrogen bond environment between interfacial and nanoconfined water. The probability distribution functions of q are summarised in Fig. S8. Based on these, one can define three structurally distinct populations of hydrogen-bond network: bulk-like within thicker channels, interfacial water along the first hydration layer of the surface, and monolayer water as its own kind. For the monolayer case, the HB network is structurally unique as it shows two well-separated peaks in the distribution function of q, contrasting both bulk-like and interfacial water. This supports the idea of a not only reorganised but also frustrated HB network. Note that such ordering is only observed for the monolayer limit whereas a combination of bulk-like and interfacial water emerges as the separation distance increases.

To discern any effects of the confining surfaces on water’s HB structure, we compare the tetrahedral order parameters for interfacial water near graphite and hBN open surfaces in direct contact with the bulk. As shown in Fig. S8b, practically indistinguishable order distributions are revealed for both surfaces. Interestingly, graphene and hBN similarities withstand even when these surfaces are brought together forming the 2D confinement environment. As shown in Fig. S8c, the HB network in monolayer water confined in graphite/hBN channels remains indistinguishable from the symmetric graphite/graphite case thus further suggesting that, under pure confinement, surface effects are essentially negligible regardless of the topology of either hBN or graphite. In fact, the spectral features calculated for such symmetrical graphene/graphene confinement are nearly identical to the hBN/graphite system used in our measurements. The DFT spectra are summarised in Fig. S10.

The structural insights gathered so far have guided us to build a detailed picture of the water’s HB domains that reshapes into a rather discontinued network at the confined monolayer state. However, as also evidenced by the peak positions of our measured spectra, a considerable fraction of the population consists of “free” or unbound water, which we assume to be located mostly across the boundaries of such HB clusters (Fig. 3i). Thus, to come close to a complete view of the structure, we now explore the structure in terms of the orientation of the free -OH subset. From several SFG studies, free -OH groups (or -OH groups that are not donating a hydrogen bond) at interfaces are well known to be stabilised out-of-plane relative to the surface^67^. However, as we have shown, in the monolayer regime interfacial effects are rather subtle and this could directly reflect on the re-orientation of these groups. Since every -OH group oriented out-of-plane is inherently unbound to its neighbouring water molecules, we adopt the free in-plane population as the best descriptor for any unusual structuring. To dwell into this question, we applied a statistical framework to analyse the orientation of the -OH groups of the overall monolayer water population, which we correlate to their HB state. We analyse the probability of an OH group to be H-bonded as a function of its orientation angle with respect to the channel surface (namely, 90 degrees for in-plane water and 0 or 180 degrees for out-of-plane-oriented molecules). The correlation is summarised in Fig. S9.

The populations of in- and out-of-plane -OH groups in the overall monolayer water phase are nearly equal. Such arrangement already differs from the bulk, where the system distributes isotropically with all orientations uniformly present. As mentioned earlier, all out-of-plane -OH groups (α < 45 degrees and α > 135 degrees) in water’s monolayer phase are necessarily free. For the in-plane population, however, while the majority of groups is expected to be hydrogen-bonded, a significant proportion of nearly 25% remains unbound to its neighbours. This contrasts what is known for bulk water, where around 95% of all -OH groups are hydrogen bonded, and interfacial water, where free -OH groups are predominantly outwards/perpendicularly oriented. Therefore, such distinct structural feature supports the concept that simply interfacial effects do not dominate the structure in monolayer confined water phases in our systems.

To enclose the link with our experiments, the transition in the blue shift amplitudes agrees well with predictions made along the last decades^68–70^ and SFG experiments^10^. Although the latter presents the measured quantities until degrees of confinement still slightly above the monolayer limit, the three-layers threshold also stands out as a cross-over, where the spectral signal starts varying as consequence of a restructuring HB network imposed by Ångström-scale confinement. In fact, a theoretical study performed earlier by Das et al.^70^ has been recently further explored by Advincula et al.^69^ then focusing on water’s structural evolution using novel machine learning-assisted MD simulations. Interestingly, their findings also suggest that on graphene surfaces at lower degrees of confinement, the orientational ordering of water is rather similar to that found in open graphene-water interfaces, dominated by the bulk with most of the free -OH populations oriented out-of-plane at the uppermost layer. Here, however, we show that the same behaviour emerges at the water-hBN interfaces thus justifying the nearly indistinguishable spectral features. As we confine water down to bi- and monolayer regime, strong confinement effects reshape the HB network so that the interface-like ordering is supressed thus giving place to such frustrated network, explaining the strong spectral shifts observed in our measurements. Surprisingly, at the extreme monolayer case, our analysis suggests that a considerable population of nearly 25% percent of in-plane bonds are also free, contrasting what is well-known for simple interfacial systems or bulk. Our results provide insights into water’s hydrogen-bonding structure down to the extreme limit of a true monolayer. We employ a sophisticated approach where water layers are templated by van der Waals heterostructures, leveraging their combined optical properties alongside high-brightness synchrotron IR experiments, and support from a robust DFT-MD theoretical framework. We believe our findings offer a fresh perspective across multiple fields, where the long-elusive HB network and the exotic behaviours of water under extreme confinement and reduced dimensions play a role.

## Methods

### Device fabrication

Our heterostructure devices (schematics in Fig. 1a) were fabricated using the bottom up procedure similar to those described in ref. ^4,6^, from which the steps are also fully detailed in ref. ^43^. This method offers good flexibility in combining multiple 2D materials targeting a combined property of interest. For the substrate, we tested the IR radiation interaction with several materials, and we standardised bare silicon chip due to its optical inactivity and high reflectivity in the frequency range of interest. The 3 × 25 μm² feed slit was etched through the chip using their preliminary silicon nitride double-sided coating as mask. The layers were transfer via wet transfer technique starting with the positioning of the bottom hBN crystal, with thicknesses ranging between 10 and 20 nm. Further etching was conducted from the rear side of the aperture through reactive ion etching, which effectively projects the aperture across the hBN. The process was repeated for the graphite layer used as the base of the angstrom-capillaries. We then isolated graphene and graphite crystals with thicknesses varying from 1 to 22 layers to produce the spacers, which were formed by patterning them into parallel stripes ~170 nm apart using electron-beam lithography. The patterned flakes were later exposed to reactive ion etching to form the independent stripes. The spacers were cleaned thoroughly to eliminate any residues from the lithography process, and atomic force microscopy (AFM) imaging was systematically used to verify the absence of contamination before the top hBN layer were transferred. This step is crucial to prevent further exposure of the spacers to contaminations. The spacer and top-hBN stack were then transferred onto the graphite layer perpendicular to the feed slit. In each device, we have also included channels that are not connected to the feed slit for background and referencing purposes. The thickness of the top hBN layer, ranging from 100 to 200 nm, was chosen to both prevent sagging and ensure full transmission of the synchrotron IR microbeam. After full assembly, annealing cycles were carried out under Ar/H_2_ at 300 °C for 4 h and then at 400 °C for 6 hours to remove any polymer residues or other contaminants picked from the previous steps. Darkfield optical images are essential throughout the transfer steps as they reveal both the presence of contaminants and possible air-bubbles trapped between layers. A further “boosting” annealing cycle was executed at least 24 hours before the measurements for each device. The fabrication steps are summarised in Fig. S1.

### Synchrotron micro-FTIR measurements

To fill the devices with water, we used a custom-made aluminium cell with a ring-shaped in lid that is screwed in but exposes the device through its 10 mm opening (see Fig. S2). Ingrooves with well-fitted O-rings are present on both cell and lid so that the capillaries are the only opening, through which water evaporates.

Synchrotron Fourier Transform IR microscopy (micro-FTIR) measurements were performed at Diamond Light Source’s MIRIAM beamline B22. The mid-IR reflectivity spectra and hyperspectral images were collected using a Bruker Hyperion 3000 microscope coupled to a Vertex 80 V FTIR Interferometer. A cassegrain 36X magnification objective (NA = 0.5) was used for all the measurements in this study. The spatial resolution is defined by microscopy knife edge blades (slits), optimised as a trade-off between overall reflectance signal from our samples and the lateral size of the channel in our devices. To secure maximal reflectivity from the capillaries, we optimised the reflected intensity on the exposed graphite layer (an area uncovered by the top hBN). Reliable signal and high signal-to-noise ratio spectra were achieved by the acquisition via 5 × 5 μm^2^ slit-size defined at the microscope, leading to an equivalent area detected at the sample plane. The individual spectra and hyperspectral images were collected using 4 cm^−1^ spectral resolution and co-adding 532 scans per spectrum (1 minute integration time per point). The IR microscope operated in 2D scanning mode via the microscope x-y controlled sample stage, and using 3 μm step, i.e., corresponding to a circa factor 2 oversampling of the final IR images.

Background measurements were collected on the zones comprising the permanently empty capillaries which are not connected to the feed slit. As mentioned above, we ensured that these zones were sufficiently large to ensure background measurements accounting for every layer forming the capillaries and not water. For the high-resolution hyperspectral images, background measurements were collected after every 10 points to prevent artefacts from any experimental fluctuations. Besides the reference spectra collected from the unconnected capillaries, all devices were measured in their initial dry state before loading onto the cell. The measurement assembly and optics are schematically represented in Fig. S2. All synchrotron measurements have been reproducible across the experimental sessions (several synchrotron beam-times).

### Data analysis

Figure S4 frames a comparison between two sample spectra on the feed slit area, from where the bulk signal is generated, and the capillaries zones. Due to the flatness and continuous stacking configuration along the area of interest, a reliable signal-to-noise ratio was maintained across the measurements. We observed, however, that the only feature affecting the signal-to-noise ratio along the stretching region was the thickness of the top hBN layer. Such effect is evident in the sample spectra displayed in Figs. 1, 2 and S5, where the top hBN thicknesses are indicated. We discard the noisy and uninfluential spectral regions (ω < 500 cm-1 and ω > 3800 cm-1). As our work focuses on the stretching modes, we have restricted the analysis the spectral window between 3000 cm^−1^ and 3800 cm^−1^. The range of interest are showed in Figs. S3b, c.

Over post-processing of the spectral data can be a critical problem in spectroscopy, as may results in over- or misinterpretation of the band features and related physical phenomena. Accordingly, we avoided commonly used tools such as denoising, atmospheric correction (i.e., subtraction by fitting) of CO_2_ bands and moisture level in our data treatment. At data acquisition, the systematic background update during the collection IR map or spectra has experimentally confirmed to be very effective in avoiding artefacts over the spectral range of interest. H_2_O stretching mode lies within the IR microscope filter detection range, which cut off is above ca. 3900 cm^−1^. We thus used the simple rubber band method over the stretching band, which is suitable for our spectral signal-to-noise ratio and just relies on the lowest points of the spectra at the two extremes of the region.

### DFT-MD calculations

We performed DFT-MD simulations of water confined between graphene and hBN as well as in symmetrical graphene-graphene confinement at three different confinement lengths L, defined as the separation of the two confining sheet atom centers of L = 0.7 nm, L = 1.4 nm, and L = 3.4 nm in the NVT ensemble. For both surface combinations, the lateral box dimensions were 2.71 × 2.98 nm^2^, 2.71 × 2.98 nm^2^ and 1.97 × 2.13 nm^2^ and the boxes contain 98, 268 and 416 water molecules, respectively. The number of water molecules in each system was chosen so that the chemical potential of the water molecules in symmetrical graphene/graphene confinement is equal to that of bulk water, as determined from force-field MD simulations conducted in earlier work^71^. All DFT-MD simulations were conducted in the CP2K/7.1 software suite, using the BLYP exchange correlation function with Grimme’s D3 dispersion correction, GTH pseudopotentials in combination with the DZVP-SR-MOLOPT basis set^72–77^. The plane wave expansion of the electronic density was truncated at 400 Ry. Simulations were conducted at 300 K, employing the CSVR thermostat^78^ with a time step of 0.5 fs for at least 75 ps each. Prior to production runs, all systems were pre-equilibrated with force-field MD simulations in the GROMACS/2023 software suit. To extract absorption spectra, the total dipole moment of the simulation box was evaluated using a periodic definition of the position operator, as implemented in CP2K. The total polarisation is unique only modulo a period that depends on the lattice vectors. A continuous polarisation trajectory was reconstructed by assuming that the polarisation changes between successive MD steps were smaller than half of this period. Absorption spectra were calculated according to the anisotropic Green-Kubo relations that were derived in ref. ^50^.

1 $${\chi }_{\parallel }\left(\omega \right)=\frac{{\phi }_{\parallel }\left(0\right)+i2\pi \omega {\widetilde{\phi }}_{\parallel }^{+}\left(\omega\right)+\,{A}_{\parallel }}{2{\varepsilon }_{0}{k}_{B}T{V}_{{eff}}}$$

2 $$\frac{{\chi }_{\perp }(\omega )}{{\chi }_{\perp }(\omega )+1}=\frac{{\varPhi }_{\perp }(0)+i2\pi \omega {\widetilde{\varPhi }}_{\perp }^{+}(\omega )+{A}_{\perp }}{{\varepsilon }_{0}{k}_{B}T{V}_{{eff}}+\left({\varPhi }_{\perp }(0)+i\omega 2\pi {\widetilde{\varPhi }}_{\perp }^{+}(\omega )+{A}_{\perp }\right){V}_{{eff}}/{V}_{{PBC}}}$$

Here, ${\varPhi }_{\alpha }(t)=\left\langle {M}_{\alpha }(0)\cdot {M}_{\alpha }(t)\right\rangle$ is the autocorrelation function of the polarisation M(t), with $\alpha=\Vert,\perp$ and we defined a one-sided Fourier-transform ${\widetilde{f}}^{+}(\omega )={\int }_{0}^{\infty }{\rm{d}}t{e}^{i\omega t}f(t)$. ε_0_ denotes the permittivity of vacuum, k_B_ is the Boltzmann constant, and T the absolute temperature. V_eff_ refers to the normalisation volume, which we chose to be defined by the lateral box dimension multiplied with the sheet separation L, for each individual system. V_PBC_ on the other hand is the volume of the periodic box employed in the DFT-MD setup. In our case, the perpendicular box length is chosen 3 times as large as the sheet separation. ${A}_{\Vert /\perp }$ is the electronic polarizability of the system, which appears in Eq.(1)-^(2^) due to the Born-Oppenheimer approximation. It is evaluated by taking 400 representative snapshots from each MD simulation and conducting single point energy calculations in the presence of explicitly applied external electric fields, with field strengths E_ext_ = 0.512 V/nm. The electronic polarizability is determined with a finite difference scheme ${A}_{\Vert /\perp }=\left\langle \frac{{M}_{\Vert /\perp }\left({E}_{\Vert /\perp }^{{ext}}\right)-{M}_{\Vert /\perp }(0)}{{E}_{\Vert /\perp }^{{ext}}}\right\rangle$ from the difference of the polarisation in the presence and absence of an electric field. All spectra are smoothed with a Gaussian Kernel in the frequency domain with a constant width of 0.4 THz. Reference bulk water data is incorporated from an earlier work [4], calculated at the same DFT-MD level of theory.

### Analysing the order extent of the water phases under Ångström- and nanoconfinement and at the channel’s interfaces

We have calculated the orientational tetrahedral order parameter introduced by Errington & Debenedetti^65^, which has been recently adapted by Wang et al.^66^ to better characterise the structure of interfacial water in terms of the angles between neighbouring water molecules. The local structure of the hydrogen bonding network is quantified by the orientational tetrahedral order parameter q, which is based on the angles ψ_jk_ that are formed between the vectors connecting oxygen atoms among the nearest neighbouring water molecules j and k.

We then compute the order parameter to take the undercoordination of purely confined and interfacial water molecules into account, according to Wang et al.^66^:

3 $$q=\left\{\begin{array}{cc}1-{c}_{N}\mathop{\sum }\limits_{j=1}^{N-1}\mathop{\sum }\limits_{k=j+1}^{N}{\left(\cos {\psi }_{{jk}}+\frac{1}{3}\right)}^{2},& \,\mathrm{if}\,N\ge 2,\\ 0\hfill & \,\mathrm{if}\,N < 2,\end{array}\right.$$

With c_N_ = 9/(2 N(N-1)), where N is the number of neighbours of a water molecule found below a cutoff radius of r_OO_ <r_c_ = 3.19 Å. This cutoff radius results into an average of 4 nearest neighbouring molecules in our bulk water simulation. In short, if q has a value of 1, the hydrogen bond structure would be perfectly tetrahedral. If it takes a value of 0, it has no order at all corresponding to an ideal gas.

## Supplementary information

Supplementary information

Description of Additional Supplementary Information

Supplementary Data 1

Supplementary Data 2

Transparent Peer Review file

## Source data

Source Data

## Data Availability

Source data of all figures and Extended Data Figs. that support the findings of this study are provided with this paper. The data corresponding to the DFT-MD simulation trajectories in this study are available along with the input files as Supplementary Data 1. Source data are provided with this paper.
