# Supplementary material for: Sub-diffractional infrared absorption of two-dimensional water
Source: Nat Commun. 2026 May 14;17:6430. doi: 10.1038/s41467-026-72629-9 (PMC13376428; doi:10.1038/s41467-026-72629-9)
Supplement: Supplementary file 1 — Supplementary information [file 41467_2026_72629_MOESM1_ESM.pdf]

Supplementary Information

Sub-diffractional Infrared Absorption of Two-dimensional Water

Marcos V. Surmani Martins[1,2], Hiran Jyothilal[1,2], Maximilian R. Becker[3], Ravalika Sajja[1,2], Ashok Keerthi[2,4,5], Roland R. Netz[3], Gianfelice Cinque[6,7], Boya Radha[1,2,5*]

[1] *Department of Physics & Astronomy, The University of Manchester, Manchester, UK, M13 9PL*

[2] *National Graphene Institute, The University of Manchester, Manchester, UK, M13 9PL*

[3] *Fachbereich Physik, Freie Universität Berlin, 14195 Berlin, Germany*

[4] *Department of Chemistry, The University of Manchester, Manchester, UK, M13 9PL*

[5] *Photon Science Institute, The University of Manchester, Manchester, UK, M13 9PL*

[6] *Diamond Light Source Ltd., Chilton-Didcot, Oxfordshire, UK*

[7] *Department of Engineering Science, The University of Oxford, OX13PJ, UK*

Device fabrication

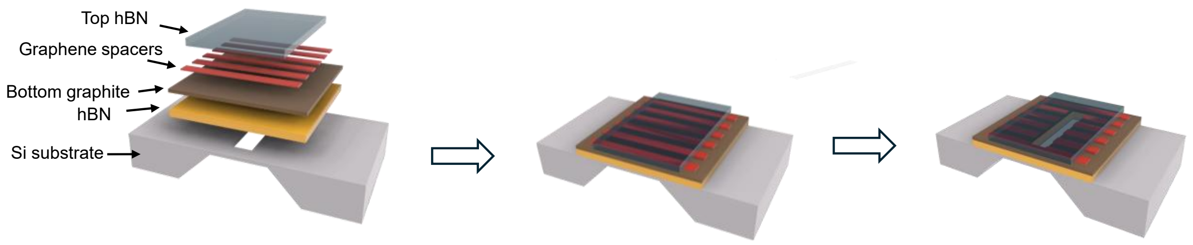

Figure S1. Device fabrication steps. The schematics summarises the nanofabrication steps used to build our devices. The heterostructure is formed *via* a transfer method following the protocol described in detail in (*1*). Once the stack is formed and positioned onto the feed slit opened through the Si substrate, dry back-etching is used to open the channels (water inlet).

Synchrotron micro-FTIR measurements

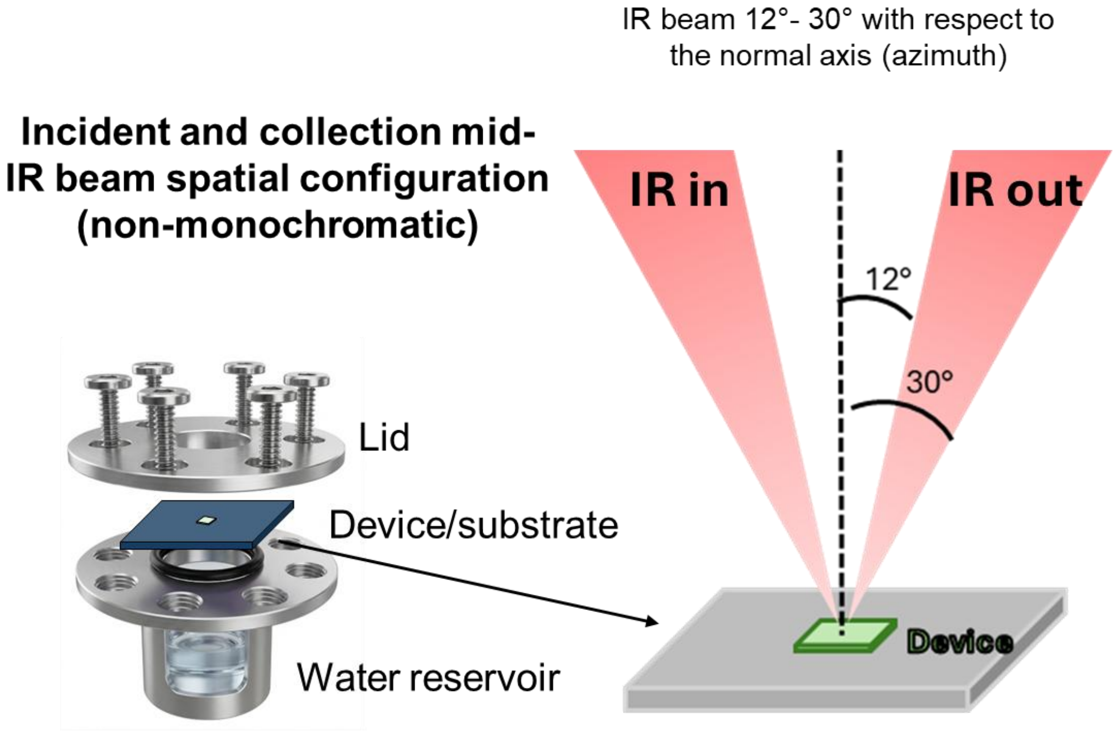

Figure S2. Measurement assembly. Sample positioning and geometries forming the optical path of the IR synchrotron beam over the sample. The full width at half maximum of the incoming SR microbeam is ca. 15 μm FWHM via a 36X objective, then the microscope slits on the outcoming beam define the $5x5um^2$ probed area at the sample plane.

Data analysis

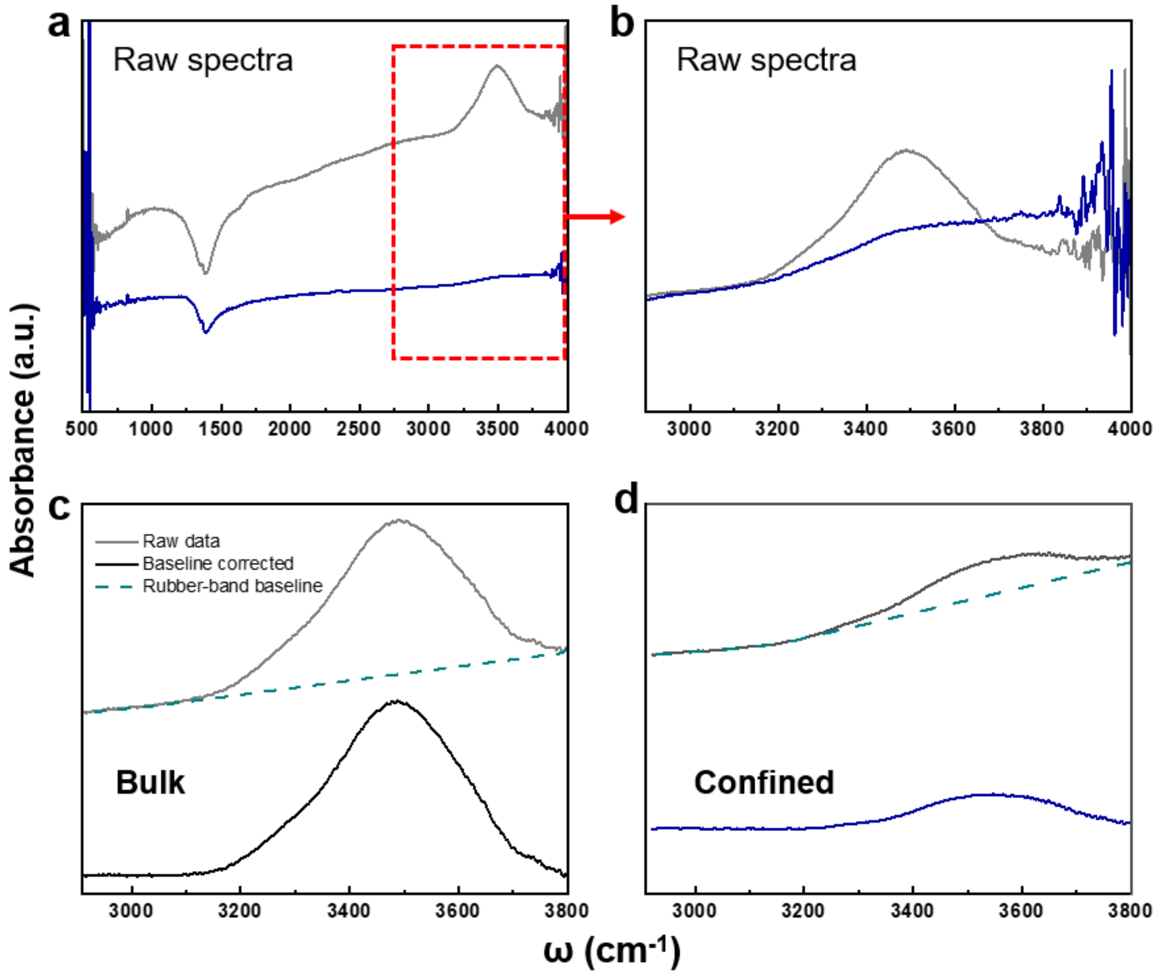

Figure S3. Data analysis and baseline correction. (a) Two sample spectra from the bulk collected at the feed slit (grey) and the capillaries zone (dark blue) in their raw format as generated from the measurement. (b) The raw form of the stretching mode range of interest as zoomed into the red dashed box in indicated in “a”. (c, d) Comparison between raw and baseline-corrected data over the -OH stretching range using the rubber band method.

Effect of top hBN thickness on band positions.

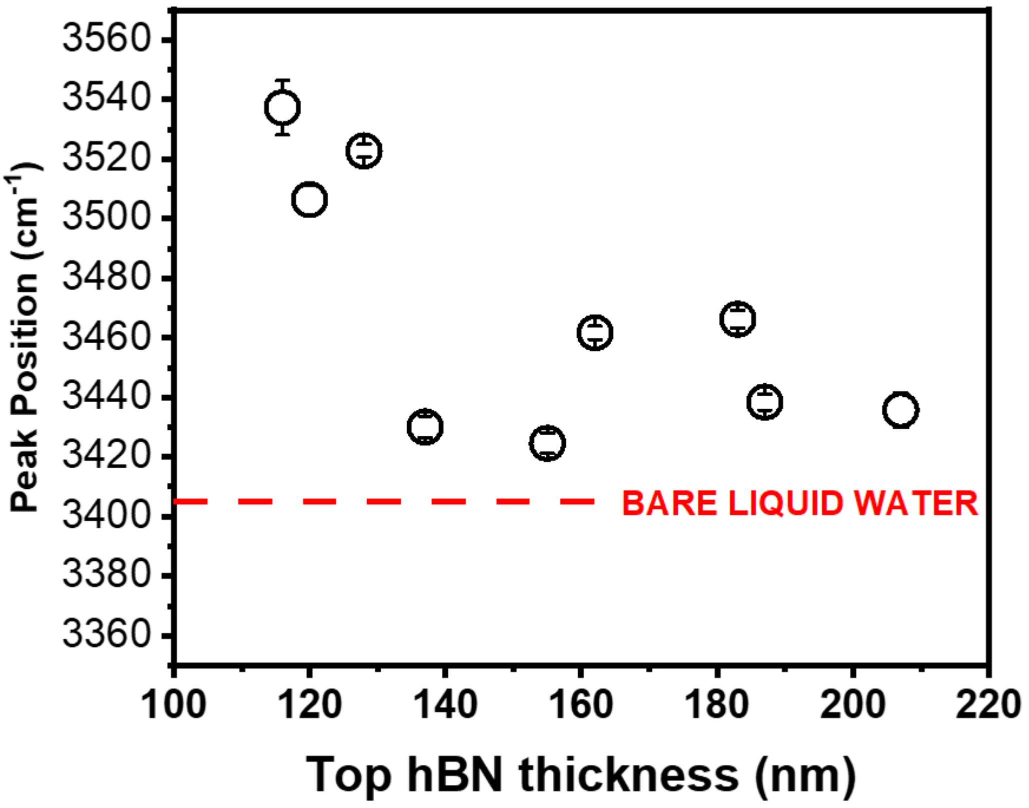

Figure S4. Effects of the top hBN layer on the peak position for the bulk water as measured on the feed slit of various devices. Data shows averaged peak positions (circles) measured along the X-Y positions over the feed slit area on the hyperspectral dataset. Each point corresponds to a single device, measured at multiple positions on the feed slit. The spectral populations for the error estimation are the number of pixels accommodated along the 25 μm long feed-slit, kept between 8 and 12 for measured hyperspectral maps on each device. For point spectra measured on control samples (a single hBN flake directly transferred over the feed slit), the errors are from 2 and 3 spectra at 120 nm and 162 nm, respectively. The thickness values here were obtained from AFM measurements of the top flake. The red dashed line represents the peak position measured for plain bulk liquid water, without hBN covering it. Data are presented as mean values ± standard error of the mean (SEM).

Sample spectra from various channel heights (complementary data to Figs. 1 & 2)

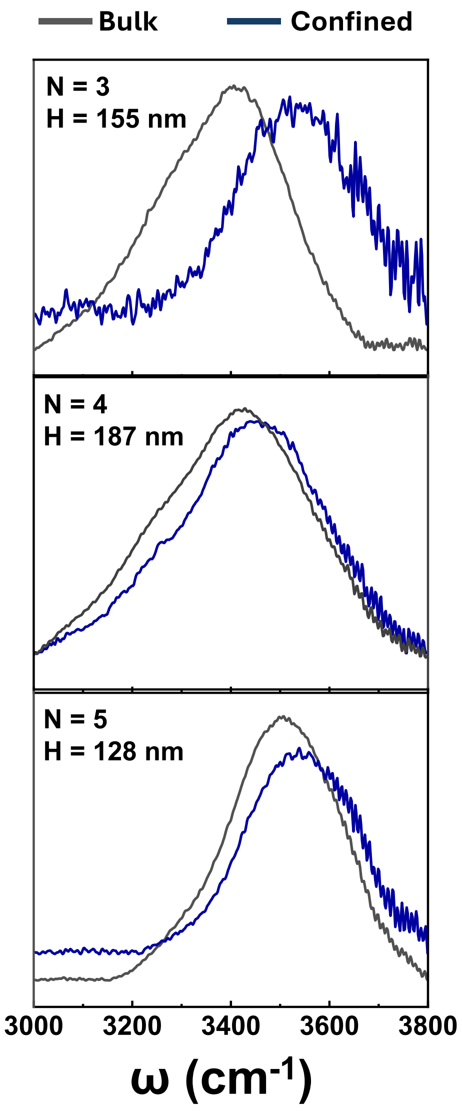

Figure S5. Blue-shift measured at different capillary heights. Water spectra complementary to figures 1 and 2 in the main text reveal the shifts caused by confinement and interfacial effects in the capillaries. All data here are compared with the bulk signal measured on the feed slit of the respective device thus already accounting for the effects of the top hBN layer.

Bending mode

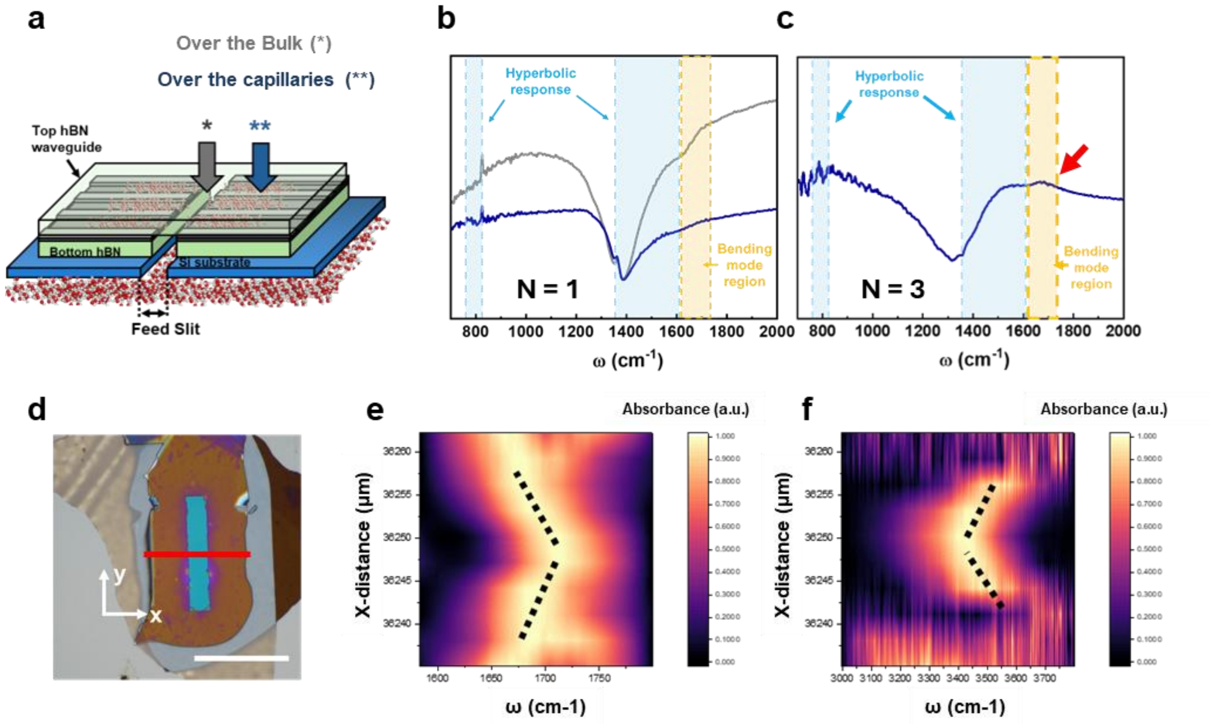

Figure S6. A glimpse of the bending mode. (a) Schematics showing the different locations where the spectra were measured. (b,c) hBN-related bands observed below 2000 cm^{-1} for a monolayer device "b" and for a 3-layered device in "c". The hyperbolic response result in strong reflectivity at ~ 1360 cm^{-1}. The bending mode starts to become evident for N ≥ 3, as indicated by the red arrow. In both cases strong damping is observed for the bulk. The blue-shaded regions are related to hyperbolic response of hBN, namely the lower and upper Reststrahlen bands. The latter overlaps the region expected for the water bending mode, which is indicated by the yellow-shaded region. For capillary thicknesses below 3 graphene layers (N<3), the bending mode as a positive peak turns almost indistinguishable. (d) Optical image of a nanochannel device with 3-layer graphene spacer, scale bar is 25 μm (e) Spectral distribution for water's bending mode along the red line indicated in "d". We note that the bending mode becomes evident for capillary heights above 3 graphene layers and shows red shifts. (f) Spectral distribution for the stretching mode at the same positions indicated in "d", revealing the blue-shifts described in this study. Dotted lines are displayed in "e" and "f" as guide to the eye.

Insights into the hydrogen-bonding network structure from DFT-MD simulations

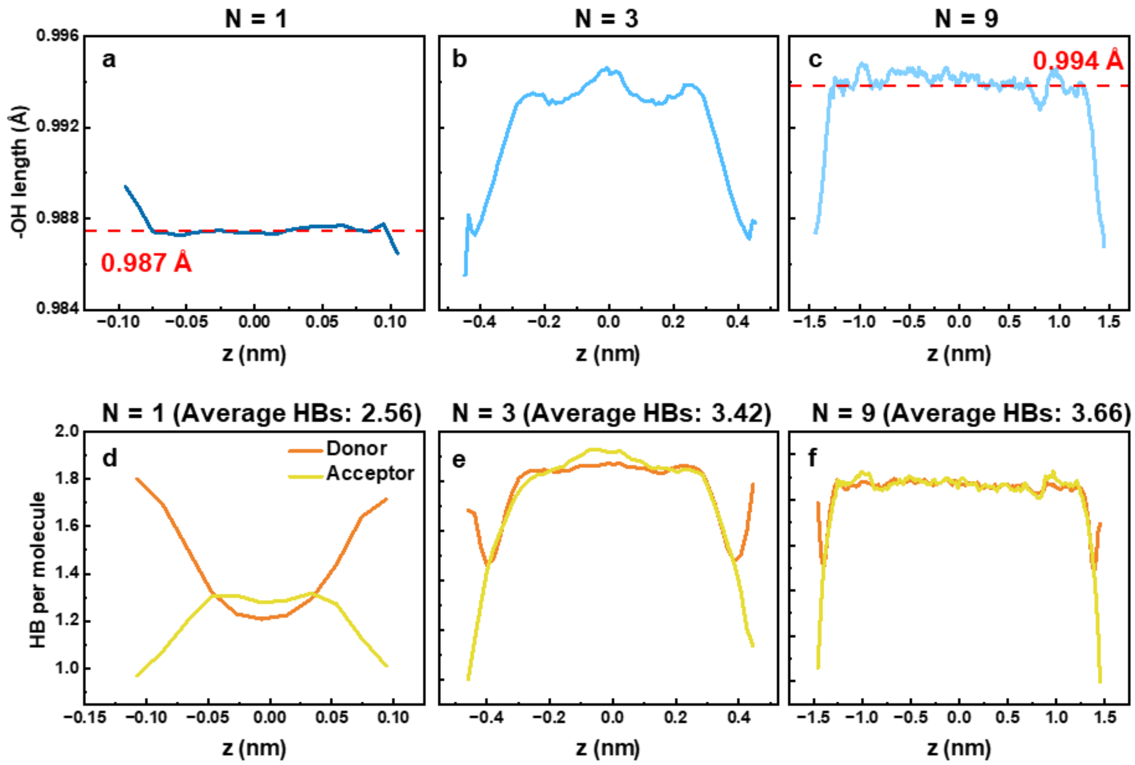

Figure S7. Predicted hydrogen bonding structure. (a-c) Average -OH bond length at different confinement conditions. The -OH bond length shrinks towards the extreme monolayer regime. (d-f) Average number of hydrogen bonds per water molecule for different channel heights. To identify a hydrogen bond, we use the standard geometric criterion shown in the main text of an O-O distance smaller than 3 Å and a HDA angle of less than 30 degrees. A network of less interconnected water molecules is favoured as confinement produces a two-dimensional configuration. Such scenario is in good agreement with the frequencies and peak shifts measured here.

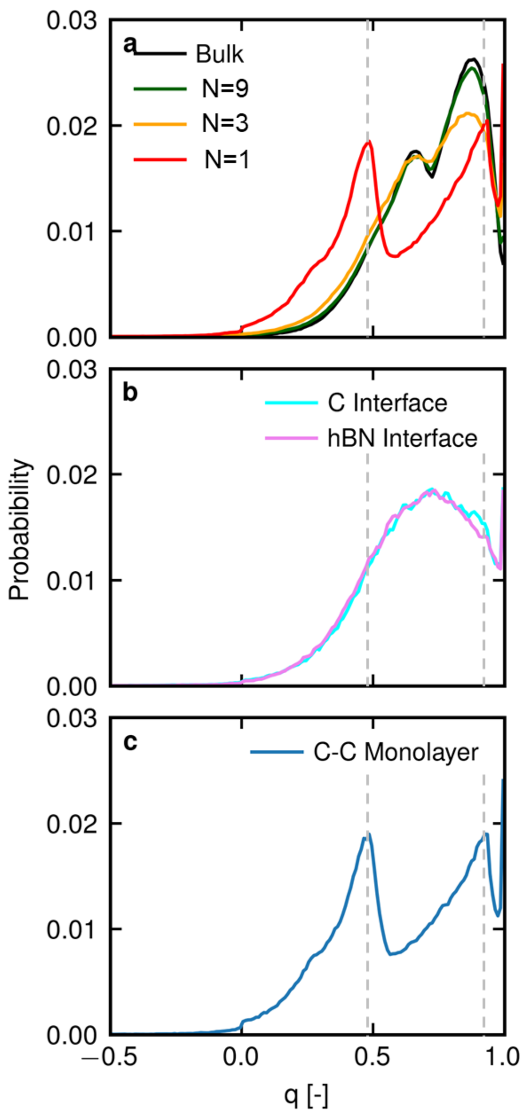

Figure S8. Ordering of the hydrogen bonding structure. (a) Probability density distribution of the order parameter q for bulk (black) and water confined between hBN and graphene sheets at different levels of confinement with N=1, 3, and 9. (b) Order distribution for interfacial water (thickness, 3.4 nm) on graphene (cyan) and hBN (pink) open surfaces. (c) Order distribution for monolayer water confined between graphene two sheets. The dashed lines are guide to the eye making the position of two distinct peaks emerging at the extreme monolayer case for both hBN/graphene and graphene/graphene confinement.

Orientation of free water molecules

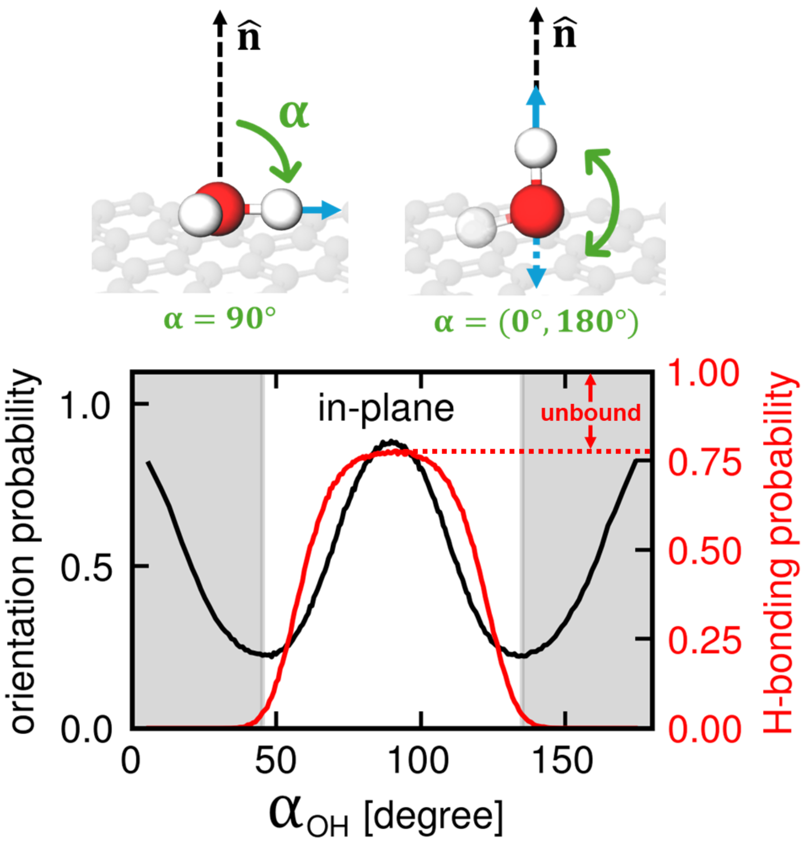

Figure S9. Orientation distribution of water molecules in the monolayer phase. The schematics in the upper panel represent the orientation condition (α) with respect to the vector $\hat{n}$, normal to the channel surface. The analysis obeys the condition that all the water molecules oriented out-of-plane ($\boldsymbol{\alpha}$ = $[\mathbf{0}°, \ \mathbf{180}°]$) are not part of the H-bonding network and are thus considered free. The black curve indicates the overall orientation distribution with nearly equal probabilities for in- and out-of-plane OH groups, whereas the red curve indicates the probability of a respective OH group to participate in hydrogen bonding. For the latter, the complement indicated by the dashed red line reveals that around 25% of the in-plane water molecules are rather free (disruptive sites), which contrasts with what is known for the bulk or at interfaces.

Monolayer water confined between graphene layers – symmetrical confinement environment.

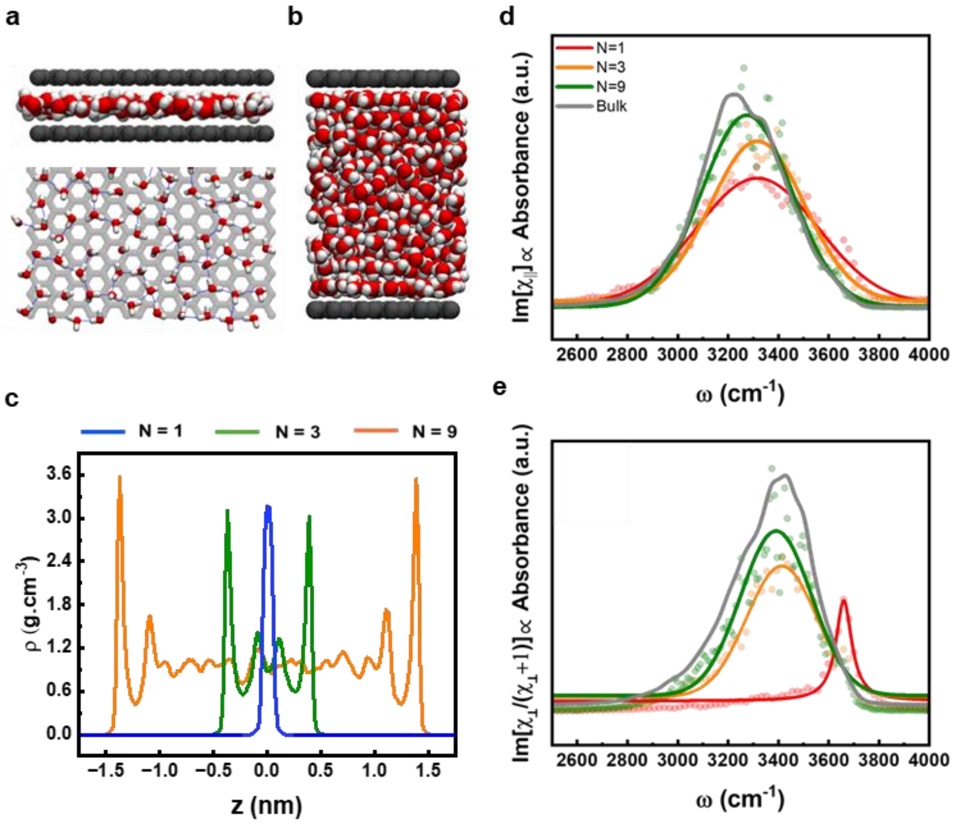

Figure S10. Two-dimensional water structure confined between two graphene sheets. (**a,b**) DFT snapshots of the collective structure for the two extreme cases in our study: monolayer and 22 layers bulk-like confined water between graphene layers. (**c**) Calculated mass density profile along the normal (out-of-plane) direction z. (**d,e**) Calculated in-plane (*d*) and out-of-plane (*e*) response of the –OH stretching bands for different confined water thicknesses based on N. The total absorption values are proportional to the imaginary terms of the in-plane response function ($\chi_{\parallel}$) and out-of-plane response function [$\chi_{\perp}/(\chi_{\perp}+1)$], where $\chi_{\parallel}$ and $\chi_{\perp}$ are the respective electric susceptibilities in each case.

Raman measurements

All Raman measurements were performed using a Renishaw inVia microscope setup with 325 nm as excitation wavelength. The measurements were performed using a UV 40X objective with the device mounted on the water reservoir similar to that used in for the micro-FTIR measurements and indicated in Fig. S2. The spectra were collected in extended/continuous mode with 10 seconds integration time and 10 times accumulation for each location along the line scan.

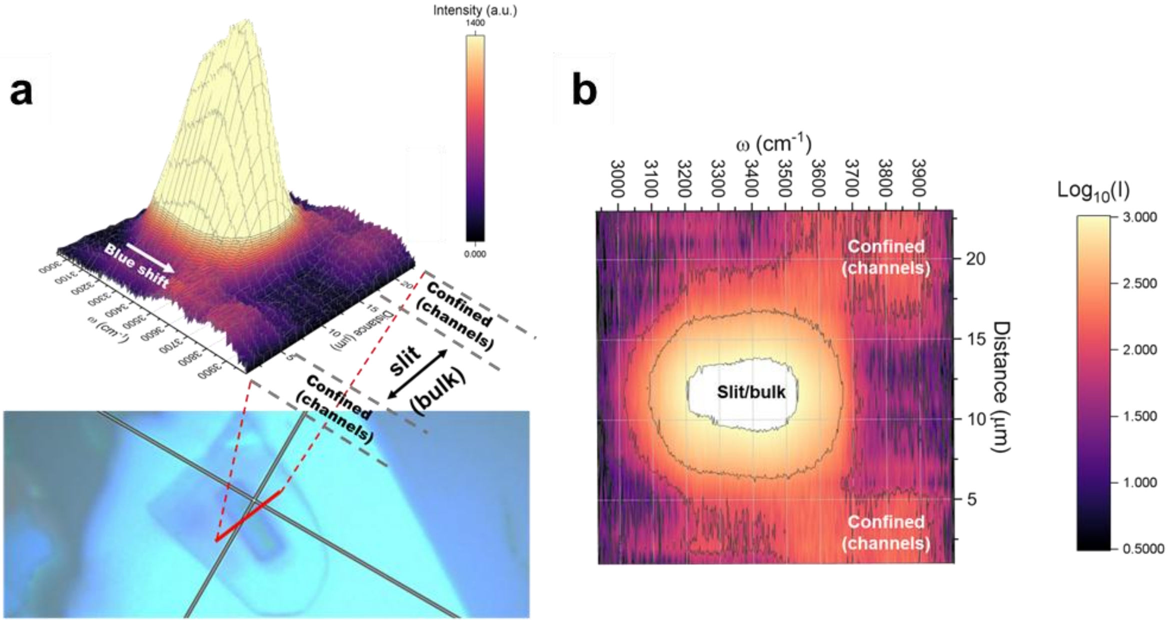

Figure S11. Raman validation of the band shifts observed for water under extreme confinement in the 2D capillaries. (a) Three-dimensional Raman spectral distribution of water stretching modes in linear scale measured along the red line extending across the feed slit (bulk) and displayed in the optical image. The blue shift is evidenced and indicated by the white arrow. The measurements here were performed at 325 nm wavelength on a device with hBN top and bottom walls. We opted for not having the graphite layer as its D" band at ~3250 cm^{-1} would overlap the water band of interest. The power density needed for a Raman spectrum can be orders of magnitude (10-100 mW) higher than IR (synchrotron IR at the sample ~1 mW). Thus, a 8-layers thin channel device (N=8) was used for a reliable signal-to noise ratio as Raman scattering cross section is very small (2 photon process), and water has a low polarizability. (b) 2D spectral distribution of the data in "a" presented in logarithmic scale.
