# Supplementary material for: Sub-diffractional infrared absorption of two-dimensional water
Source: Nat Commun. 2026 May 14;17:6430. doi: 10.1038/s41467-026-72629-9 (PMC13376428; doi:10.1038/s41467-026-72629-9)
Supplement: Supplementary file 2 — Description of Additional Supplementary Information [file 41467_2026_72629_MOESM2_ESM.pdf]

Description of Additional Supplementary Files

File Name: Supplementary Data 1
Description: File descriptions of - Molecular dynamics trajectories and input files for asymmetric hBN-graphene channels and symmetric graphene-graphene channels.

File Name: Supplementary Data 2
Description: Molecular dynamics trajectories and input files for asymmetric hBN-graphene channels and symmetric graphene-graphene channels.
