# Supplementary material for: Sub-diffractional infrared absorption of two-dimensional water
Source: Nat Commun. 2026 May 14;17:6430. doi: 10.1038/s41467-026-72629-9 (PMC13376428; doi:10.1038/s41467-026-72629-9)
Supplement: Supplementary file 3 — Supplementary Data 1 [file 41467_2026_72629_MOESM3_ESM.docx]

**Supplementary Data 1**

**Description:** Molecular dynamics trajectories and input files for asymmetric hBN-graphene channels and symmetric graphene-graphene channels

**Asymmetric hBN-graphene channels:**

**first_frame_hBN_graph_N=1.xyz**: Initial molecular configuration of water confined in monolayer (one graphene-layer thin channels, N=1). The channels are comprised of hBN top layer and graphene bottom layer.

**last_frame_hBN_graph_N=1.xyz**: Final molecular configuration of water confined in monolayer (one graphene-layer thin channels, N=1). The channels are comprised of hBN top layer and graphene bottom layer.

**hBN_graph_N=1.inp**: Input files for molecular trajectory between initial and final configurations in monolayer (N=1) asymmetric hBN-graphene channels.

**first_frame_hBN_graph_N=3.xyz**: Initial molecular configuration of water confined in three graphene-layers thin channels, N=3. The channels are comprised of hBN top layer and graphene bottom layer.

**last_frame_hBN_graph_N=3.xyz**: Final molecular configuration of water confined in three graphene-layers thin channels, N=3. The channels are comprised of hBN top layer and graphene bottom layer.

**hBN_graph_N=3.inp**: Input files for molecular trajectory between initial and final configurations in three-layer thin (N=3) asymmetric hBN-graphene channels.

**first_frame_hBN_graph_N=9.xyz**: Initial molecular configuration of water confined in nine graphene-layers thin channels, N=9. The channels are comprised of hBN top layer and graphene bottom layer.

**last_frame_hBN_graph_N=9.xyz**: Final molecular configuration of water confined in nine graphene-layers thin channels, N=9. The channels are comprised of hBN top layer and graphene bottom layer.

**hBN_graph_N=9.inp**: Input files for molecular trajectory between initial and final configurations in nine-layer thin (N=9) asymmetric hBN-graphene channels.

**Symmetric graphene-graphene channels:**

**first_frame_graph_N=1.xyz**: Initial molecular configuration of water confined in monolayer (one graphene-layer thin channels, N=1). The channels are comprised of graphene as both top and bottom layer presented in Figure S10.

**last_frame_graph_N=1.xyz**: Final molecular configuration of water confined in monolayer (one graphene-layer thin channels, N=1). The channels are comprised of graphene as both top and bottom layer presented in Figure S10.

**graph_N=1.inp**: Input files for molecular trajectory between initial and final configurations in monolayer (N=1) symmetric graphene-graphene channels presented in Figure S10.

**first_frame_graph_N=3.xyz**: Initial molecular configuration of water confined in three graphene-layers thin channels, N=3. The channels are comprised of graphene as both top and bottom layer presented in Figure S10.

**last_frame_hBN_graph_N=3.xyz**: Final molecular configuration of water confined in three graphene-layers thin channels, N=3. The channels are comprised of graphene as both top and bottom layer presented in Figure S10.

**graph_N=3.inp**: Input files for molecular trajectory between initial and final configurations in three graphene-layers thin (N=3) symmetric graphene-graphene channels presented in Figure S10.

**first_frame_graph_N=9.xyz**: Initial molecular configuration of water confined in nine-layers thin channels, N=9. The channels are comprised of graphene as both top and bottom layer presented in Figure S10.

**last_frame_graph_N=9.xyz**: Final molecular configuration of water confined in nine-layers thin channels, N=9. The channels are comprised of graphene as both top and bottom layer presented in Figure S10.

**graph_N=9.inp**: Input files for molecular trajectory between initial and final configurations in nine-layers thin (N=9) symmetric graphene-graphene channels presented in Figure S10.
