# Supplementary material for: Sub-diffractional infrared absorption of two-dimensional water
Source: Nat Commun. 2026 May 14;17:6430. doi: 10.1038/s41467-026-72629-9 (PMC13376428; doi:10.1038/s41467-026-72629-9)
Supplement: Supplementary file 5 — Transparent Peer Review file [file 41467_2026_72629_MOESM5_ESM.pdf]

nature portfolio

Peer Review File

Sub-diffractional infrared absorption of two-dimensional water

Corresponding Author: Professor Boya Radha

This file contains all reviewer reports in order by version, followed by all author rebuttals in order by version.

Version 0:

Reviewer comments:

Reviewer #1

(Remarks to the Author)
This is a great paper that for sure should be published. I amazed that such samples/devices can be prepared, and that one can measure IR data of monolayer water which such good signal-to-noise ratio. The result is in essence a blue-shift of the water OH spectrum, which can be reproduced in a semi-quantitative way by ab-initio MD, which in turn then can say what that shift means in terms of water structure. These is for sure very important findings from a basic research point of view

I cannot, and will not, comment on the device preparation since that is not my field of expertise. I feel qualified though to comment of the IR-spectroscopic aspect of the paper. I that regard, I have one major concern:

(1) While it of course so indeed that a blue shift is caused by weaker hydrogen bonding, weaker hydrogen bonding typically also results in more narrow bands, which however is not observed. SFG spectra are the best example in that regard, which always show a narrow line at around 3600 cm-1, which is assigned to the “dangling” OH without any hydrogen bond. The frequency of the N=1 case is about that frequency, hence one would essentially interpret it as non-hydrogen-bonded (and the authors indeed do), yet it is equally broad as in bulk water. I don’t understand that. There is an easy explanation of the spectral width: That is, hydrogen-bonded OH’s are much broader than typical vibrational modes because (a) the frequency is a measure of hydrogen bond strength, and (b) there is a wide distribution of hydrogen bond strengths in a disordered liquid, since every water molecule sits is a slightly different environment. However, from that argument it follows that a non-hydrogen-bonded water should be more narrow, since there cannot be any distribution of hydrogen bond strengths when there is no hydrogen bond.

(2) Related to that: The DFT results in Fig. 3f show exactly the result I mentioned above, but is essence not discussed. It furthermore shows that result only for one polarisation condition, and I don’t understand why. I believe that the experiment measures the other polarisation condition, i.e. that of Fig. 3e, which does not show that narrowing of the band, so it would be consistent with experiment, but I still miss a discussion on that.

(3) maybe related: It is mentioned that he reason for the good signal-to-noise is some signal-enhancement due to plasmonic effects. I can see that this is happening, but is also dangerous. Could it be that the frequency shifts are a result of those plasmonic effects, e.g., since the latter has a resonance that enhances mor the blue part of the water spectrum.

(4) It is said that the density of water is increased upon hydrogen bonding. How can that lead to a reduction of HB strength. I sort of missed the molecular picture behind that.

The discussions of all these aspects needs to be extended significantly, also what we learn from the DFT.

Minor comments:

I don't understand the aspect of "sub-diffractional" in the title. I don't think it is relevant for the message of the paper

Page 6, “see AFM topography in Fig 1 b,c)” should probably be Fig. 1d

In Fig. 1c, the labelling “Bulk” and “Channels” is offset relative to the lines they should describe

Page 6, “In Fig. 1f, we confirm..” should be Fig. 1g

Page 7: I thought the sentence “As the rotational quanta of energy are in the order of only a few cm-1 (~10-4 eV, primarily in the microwave-THz region of the spectrum), they rather provide valuable insights into rotational modes but offer limited information about the intermolecular forces for small molecules such as water (20). “ was pretty meaningless. Nobody in the community would be surprised to look at the OH stretch vibrations, plus, rotations are not visible anyways in solutions.

Page 8: The sentence “As shown in the representative spectrum from the monolayer case in Fig 1e, the centroid blue-shifts when under confinement.” is the very result of the paper. But it takes 2 pages after introducing the corresponding results in Fig 1 with in part not very relevant discussion (e.g.my previous point) until the authors come to that point. I would recommend restructuring the paper a little bit not to loose readers.

Page 10: I don’t understand the sentence: “Although HB does not directly influence the band shifts, it affects the lifetime of the intramolecular vibrational modes”. The very point of the paper is that the HB does affect the band shift, the experiments performed don’t measure lifetimes (it is linear spectroscopy), and the lifetime has no effect of the band shift.

Page 10: Disentangling the IR spectrum of water in the way described in the sentence “ 3140 cm-1, linked to strongly bound or ice-like water; 3220 cm-1 from tetrahedral water; 3400 cm-1 from tetramers; 3540 cm- 1 from trimers and dimers; and 3600 cm-1 from vapour-like or “free water” is highly controversial, to say the least. Certainly, I would not trust a multi-Gaussian fit to disentangle those species. In fact the authors don’t do such a fit, so why introducing that (in my opinion) very problematic assignment.

Reviewer #2

(Remarks to the Author)
This is a very interesting manuscript that opens a new avenue to experimentally probe confined water down to a single monolayer, where the properties of the H-bond network are substantially altered compared to bulk and interfacial water. The approach is sound and original, and the manuscript deals with an urgent topic of broad interest for interfacial science. In the future, similar studies could be used to learn about how the properties of the confining matrix, e.g., its topology and morphology, can be exploited to template different water H-bond networks. Therefore, I will be happy to support publication after the following remarks are addressed:

- It would be nice if the authors could provide more information on what they define as an anomalous phase of water. The decomposition into local water structure is interesting but not enough to get a comprehensive picture: how is the water H-bond network organized, especially for the single monolayer? Which kind of H-bonded pattern do the water molecules form? How does it adapt to the topology and morphology of the surface? Are the free OH groups only oriented out-of-plane (as usually observed as solid-liquid interfaces, e.g., with SFG) or also within the plane due to the high degree of confinement that frustrates the H-bond network? The DFT calculations and MD simulations could be used to provide much more information here, which I think will benefit the broad impact of the manuscript. Figure 3 already provides some of this information, and I would be interested to read more on that.

- Also, some information on the water dynamics, as well as the evaluation of the common order parameters for water, may be insightful to understand to what extent this can be defined as an anomalous phase

- I would also like to read more about the transition in the spectroscopic signature and water structure when going from 10 to 1 water layer. A deeper comparison with previous observations for confined water in the literature could be beneficial. The picture I have in mind from previous studies is that, in terms of the structure and spectroscopic response (as measured in SFG spectra), the topmost water monolayer in contact with the surface doesn't change dramatically upon increasing the degree of confinement down to 3 water layers (e.g., ref.https://www.nature.com/articles/s41467-025-62625-w). Please elaborate

Reviewer #3

(Remarks to the Author)
Referee report on manuscript NCOMMS-25-58925-T

This manuscript reports a direct experimental observation of vibrational absorption in a monolayer-confined water, which is a very relevant scientific feature. The authors employ narrow slit capillaries built from van der Waals heterostructures (graphite + hexagonal Boron Nitride) and synchrotron infrared microspectroscopy to detect OH-stretching signatures at single-layer resolution. The measurements reveal significant blue shifts consistent with a weakened hydrogen-bond network, supported by DFT-MD simulations. The data provided in this manuscript suggest that confined water forms a dense but weakly hydrogen-bonded phase, a structural regime distinct from both bulk liquid and vapour. In my opinion, this is not a truly novel statement, since previous works have observed different water phases though peculiar hydrogen-bond rearrangements. Lacking references (see below) can enlighten the readers about this point.

On novelty and impact, this work provides direct and unprecedented experimental access to the vibrational structure of monolayer water, a topic of broad interest in nanofluidics, interfacial science, and condensed matter physics. Further, DFT-

MD simulations corroborate experimental findings overall, particularly HB weakening, density increase, and anisotropy in vibrational response. On methodology, the device design combining graphite reflectivity and hBN waveguiding to amplify weak IR absorption is pioneering and it can be employed beyond this work. The use of synchrotron IR microspectroscopy avoids common heating artefacts. Finally, the authors report clear and reproducible spectral shifts with strong control measurements (empty vs filled channels, bulk vs confined).

Aspects to improve:

The study relies on centroid shifts of the -OH band. A more detailed sub-band decomposition (even constrained by simulations) could yield deeper insights into HB motifs.
On the comparison to simulations, my remark is that, whereas DFT-MD simulations model graphene confinement, the experiments use graphite + hBN. The possible differences and limitations of this approximation deserve fuller discussion. Direct comparison is not trivial.
The study focuses exclusively on graphite–hBN confinement. A discussion of how results may (or may not) extend to other hydrophobic or hydrophilic environments would strengthen the impact. Carbon nanotubes would be an excellent candidate.
The conclusion of a threefold density increase in monolayer water is based on indirect reasoning. Without direct structural probes (e.g., X-ray or neutron scattering), this remains somewhat speculative. Verify or remove this conclusion.
On references: This manuscript lacks some context on previous works related to the confinement of water in reduced dimensions and its behaviour at interfaces. Concerning the experimental side, I will mention works published by Skinner at al. (Acc. Chem. Res. 2012, 45, 1, 93–100, https://doi.org/10.1021/ar200122a ; Nature 474, 192–195 (2011). https://doi.org/10.1038/nature10173 or PNAS 104 (36) 14215-14220 (2007). https://doi.org/10.1073/pnas.0701482104) and works form Bakker et al. (Chem. Rev. 2010, 110, 3, 1498–1517, https://doi.org/10.1021/cr9001879; PNAS 102 (9) 3202-3207 (2005) https://doi.org/10.1073/pnas.0404916102) among others. Form theoretical-computer simulations, works indicating the alteration of water properties under strict confinement, such as inside carbon nanotubes, for instance, are also worth to be mentioned: Acc. Chem. Res. 2017, 50, 9, 2139–2146. https://doi.org/10.1021/acs.accounts.6b00617 ; Phys. Rev. B 63, 165430 (2001). https://doi.org/10.1103/PhysRevB.63.165430 ; Sci Rep 15, 28144 (2025). https://doi.org/10.1038/s41598-025-14266-8 ; J. Phys. Chem. C 122, 6684–6690 (2018). https://doi.org/10.1021/acs.jpcc.8b00112 ; J. Am. Chem. Soc. 2006, 128, 37, 12090–12097. https://doi.org/10.1021/ja057856u , etc. All these references (and many others) are relevant to focus the reader in the appropriate context, what is important to emphasize the novelty and relevance of the present work. This drawback is one of the issues that need to be addressed.
Improve figure clarity and streamline technical sections for accessibility.

In summary, this work addresses a long-standing challenge in water science, bridging the gap between theoretical predictions and experimental validation. It represents a significant technical and conceptual advance in the study of confined liquids. All in all it is a technically impressive and conceptually important contribution that will attract broad interest. I recommend major revision following the lines indicated above, before eventual acceptance.

Version 1:

Reviewer comments:

Reviewer #1

(Remarks to the Author)
The authors have very thoroughly addressed my concerns. I do not agree on everything (in particular the title), but they now discuss why they do what they do. I recommend publication of the paper in its present form. Again, this is a very nice paper.

Reviewer #2

(Remarks to the Author)
The authors carefully addressed my comments, and I am happy to recommend publication

Reviewer #3

(Remarks to the Author)
Dear authors,

Following the first full round of revision, I’m pleased to say all major issues have been satisfactorily resolved and the manuscript is now ready for publication. I’m particularly delighted with the changes made after considering my list of concerns.

Open Access This Peer Review File is licensed under a Creative Commons Attribution 4.0 International License, which permits use, sharing, adaptation, distribution and reproduction in any medium or format, as long as you give appropriate credit to the original author(s) and the source, provide a link to the Creative Commons license, and indicate if changes were made.
In cases where reviewers are anonymous, credit should be given to 'Anonymous Referee' and the source.
The images or other third party material in this Peer Review File are included in the article's Creative Commons license, unless indicated otherwise in a credit line to the material. If material is not included in the article's Creative Commons license and your intended use is not permitted by statutory regulation or exceeds the permitted use, you will need to obtain permission directly from the copyright holder.
To view a copy of this license, visit https://creativecommons.org/licenses/by/4.0/

REVIEWER COMMENTS

Reviewer #1 (Remarks to the Author):

This is a great paper that for sure should be published. I amazed that such samples/devices can be prepared, and that one can measure IR data of monolayer water which such good signal-to-noise ratio. The result is in essence a blue-shift of the water OH spectrum, which can be reproduced in a semi-quantitative way by ab-initio MD, which in turn then can say what that shift means in terms of water structure. These is for sure very important findings from a basic research point of view

We are very pleased with the reviewer's constructive report on our work, both valuing our findings and highlighting their fundamental relevance to the field of condensed matter physics/water science.

Besides the comments addressed below, and the changes suggested by the reviewer, we would like to highlight that the DFT-MD model is now been updated to better represent our system consisting of water confined between hexagonal boron nitride and graphite. From our simulations, we further explored the spectral signature of water from a structural point of view and introduced a detailed analysis of the hydrogen bonding network and the structure in terms of tetrahedral ordering.

I cannot, and will not, comment on the device preparation since that is not my field of expertise. I feel qualified though to comment of the IR-spectroscopic aspect of the paper. I that regard, I have one major concern:

(1) While it of course so indeed that a blue shift is caused by weaker hydrogen bonding, weaker hydrogen bonding typically also results in more narrow bands, which however is not observed. SFG spectra are the best example in that regard, which always show a narrow line at around 3600 cm-1, which is assigned to the "dangling" OH without any hydrogen bond. The frequency of the N=1 case is about that frequency, hence one would essentially interpret it as non-hydrogen-bonded (and the authors indeed do), yet it is equally broad as in bulk water. I don't understand that. There is an easy explanation of the spectral width: That is, hydrogen-bonded OH's are much broader than typical vibrational modes because (a) the frequency is a measure of hydrogen bond strength, and (b) there is a wide distribution of hydrogen bond strengths in a disordered liquid, since every water molecule sits is a slightly different environment. However, from that argument it follows that a non-hydrogen-bonded water should be more narrow, since there cannot be any distribution of hydrogen bond strengths when there is no hydrogen bond.

This is certainly a very important point to the big picture of our work, and we thank the reviewer for highlighting it.

A brief discussion on the terminology used:

When framing the paper, the authors had mindful discussions regarding the terminology to better describe the water state from a hydrogen-bonding perspective. In the previously submitted version, the term "weaker hydrogen-bonds" has been chosen as one could primarily infer it from our

observables, i.e. the centroid blue shifts. However, we do agree that the matter is more complex from a local-structure point of view, for which the term “fewer hydrogen-bonds” (per water molecule) would better fit to describe it. The DFT snapshot in Fig. 3h in the main text frames such state in which a disruptive network (represented by the blue-dashed links) emerges as a consequence of the confinement. Non-hydrogen-bonded water would thus form a fraction of such frustrated network. From our simulations, we obtain that the average number of hydrogen-bond per water molecule decreases significantly in the monolayer case (2.56, Fig. S7d) when compared with the tetrahedral arrangement well-known for the bulk (3.66, Fig. S7f).

The same picture can be formed from a geometric analysis. We are now complementing Fig. 3 with an additional analysis on the hydrogen-bonding angle distribution. While the 30 degrees geometric criterion prevails for the bulk and ‘bulk-like’ case in thicker channels, a wing indicating wider hydrogen-bonding angles emerges towards the limiting monolayer case.

Considerations on the spectral width:

We are glad that the reviewer brought up this comment regarding the spectral width. In fact, we believe that not only the hydrogen bonding structure, but also the dielectric response of water at such extreme confinement would influence the band width. Please, find below the points discussed separately for clarity:

i. Hydrogen-bonding structure: Upon the reviewer’s kind observation, we do recognise that the term “vapour-like” was rather misused in view of the actual spectral features revealed in our measurements. In such confinement conditions, we do not only find “free water” but rather a disordered liquid structure. Thus, we should also account for contributions from the populations of H-bonded molecules with vibrational frequencies spreading towards lower wavenumbers as part of such frustrated hydrogen-bonding network (with 2 to 3 HB per water molecule as per our model), thus broadening the overall stretching band. The wording choice was initially motivated by the position of the band centroid, which falls at frequency values comparable with that of vapour or free water, yet accounting for the contributions of the sub-modes at lower frequencies.

Interestingly, in literature, experimental observations on water confined in carbon nanotubes revealed a vibrational response comparable with our findings (https://arxiv.org/pdf/2402.17989). Although one can relate to these systems as a one-dimensional analogue of our channels, their results also suggest the emergence of a disrupted hydrogen-bonding network, as the carbon nanotube diameters reaches its limiting value of 1.4 nm. These experiments were performed using vibrational electron energy loss spectroscopy (vEELS), also a totally different technique where the vibrational modes of water were rather assessed through the momentum transfer between electrons in a transmission electron microscope and the water phase confined in a carbon nanotube placed in the vicinities of the beam. Interestingly, despite being a non-optical and non-linear vibrational technique, the centroid shifts and band widths are comparable with values observed in our experiments.

In summary, we thank the reviewer for bringing our attention to these points that are certainly relevant to the big-picture of our work. We have revisited the terminology used and after consideration the authors all agree that “fewer” rather than “weaker” hydrogen bonds per water molecule is what better describes our system at the extreme monolayer state. In this scenario, which is supported by our DFT-MD model, not only free but also H-bonded water molecules form the overall network with the contribution of sub-modes at lower frequencies thus broadening the overall stretching-mode band.

Therefore, to better reflect our system, we have rewritten the sentence as follows:

"In Fig 2, large blue-shifts appear as we scale down to a single water layer, with centroid values moving towards higher frequencies suggesting significant changes in the HB network. This effect saturates as we increase the channel thickness and the HB network is recovered towards its bulk-like configuration, where only interfacial effects remain. Fig. 2c reveals the stark contrast in the relative spectral features for a bilayer and a 22 layered (8 nm thick) capillary. Specifically, the magnitude of such blue-shifts in our data indicates that a rather disordered HB structure emerges as we scale down to the monolayer configuration, with fewer HBs donated per water molecules (in average). Such confinement-to-bulk transition agrees well with a recent theoretical study on water confined between graphene layers probed via THz and IR absorption (8). Here, with a step further into the sub-nanometre range, we experimentally validate these findings on confinement effects on the spectral response taking place at confinement thicknesses below 1.4 nm (N=4). The steep increase in the blue-shifts for N < 4 values in Fig. 2b is evident and consistent with their findings despite the use of hBN as top layer in our channels."

ii. Anisotropic dielectric properties of confined water: As our simulation suggests, the dielectric function of water and, therefore, its polarizability becomes extremely anisotropic under extreme confinement at lower dimensions. A much deeper analysis on how such effect evolves can be found in the recent study developed independently by some of the authors (https://doi.org/10.1063/5.0239693), for water layers confined between graphene sheets analysed with excitations across the THz-IR range.

For our system, such anisotropic spectral response is depicted in Fig. 3a and b. We clearly notice that even for the limiting monolayer case, the tangential response (parallel to the confinement planes) emerges at frequencies lower than that observed for the normal (out-of-plane). In our experiment, however, since **both directions are being probed at off-normal incidence** (see details in Fig. S2), the in-plane response towards lower frequencies of the stretching range depicted in Fig.3e would have noticeable influence on overall spectral width.

Interestingly, these results corroborate with recent experimental studies made on similar channels using scanning dielectric microscopy (https://www.science.org/doi/10.1126/science.aat4191, https://arxiv.org/pdf/2407.21538), where the dielectric constant and conductivity of water were measured under quasi-2D confinement. Although measured in the low frequency limit and focused the real part, their findings also indicate that the dielectric function of confined water shows a significant contrast between the in- and out-of-plane directions, with a much strong response for the former.

(2) Related to that: The DFT results in Fig. 3f show exactly the result I mentioned above, but is essence not discussed. It furthermore shows that result only for one polarisation condition, and I don't understand why. I believe that the experiment measures the other polarisation condition, i.e. that of Fig. 3e, which does not show that narrowing of the band, so it would be consistent with experiment, but I still miss a discussion on that.

We appreciate that the reviewer has pointed this out. A discussion based on the points detailed above (points ii and iii) is being added to the main text. Moreover, **we present and discuss DFT results for both polarisations**, correlating with their respective contributions to the shifts and width of the measured spectra.

Both components presented in the main text rather offers a qualitative view of such anisotropy. As discussed above, because of our off-normal incidence, we have a superposition of both components.

It is worth mentioning we initially tried combining both as a semi-quantitative approach. However, our analysis suggests that such relationship is rather more complex and deserves a deeper attention to it as the signal intensity is also directional due to the hyperbolic nature of hBN (anisotropic refractive index), which seems crucial for the enhancement. Such behaviour is known for confining the light in the THz to NIR down to sub-diffractional scales *via* resonant modes. In fact, this became a whole new study that we are currently carrying out combining both far- and near-field (nano-FTIR) to better understand local resonating effects contributing to the enhancement.

(3) maybe related: It is mentioned that he reason for the good signal-to-noise is some signal-enhancement due to plasmonic effects. I can see that this is happening, but is also dangerous. Could it be that the frequency shifts are a result of those plasmonic effects, e.g., since the latter has a resonance that enhances mor the blue part of the water spectrum.

This is a fair point, and we appreciate that the reviewer has brought our attention to it.

Interestingly, when our signal-enhancement was first observed, we initially thought about the possibility of it being mostly related to plasmons originated from the delocalised electrons on the graphite surface. We further hypothesise that such delocalised charges could even enhance the dipole moment of the confined water molecules by forming anti-mirrored charge dipoles along the graphite plane induced by the water molecules. However, we ruled this hypothesis out by performing a control experiment, where we removed the graphite layer and only hBN accommodates the channels. The spectra are presented below and shows that the enhancement is still observed along with the shifts expected for a monolayer device. However, Fabry-Perot fringes emerge from the resonance between the top and bottom semi-transparent hBN layers across the channels zone. As the bulk signal only accounts for the top layer over the feed micro-hole, such effect is not observed. Moreover, we notice that the reflected intensity is suppressed by almost an order of magnitude, implying that the highly reflective graphite layer is not only crucial for a good signal-to-noise ratio, but also to eliminate the Fabry-Perot cavity effect.

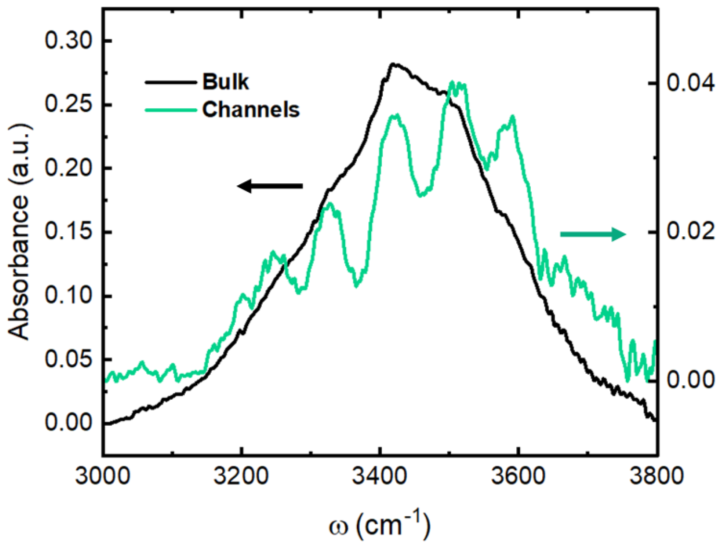

Figure R1: Control experiment to understand the contribution of the graphite reflective layer: Water spectra collected for a monolayer device (N=1) without the graphite layer, where only the top and

bottom hBN comprise the heterostructure. Both spectra were collected on the same manner as the data presented in the main text.

(4) It is said that the density of water is increased upon hydrogen bonding. How can that lead to a reduction of HB strength. I sort of missed the molecular picture behind that.

We do agree with the reviewer's concern that this is not a trivial correlation. Unfortunately, since we are dealing with volumes at sub-zeptoliter scales, an accurate measurement of the actual density remains an experimental limitation as it falls far below the detection limit of any current technique. However, structural or vibrational observables, even if indirect, can provide essential inputs for computational methods from which mass-dependent properties can be estimated. Here, as our spectral features show high consistency with an early work developed independently by two of our authors, we adapted their previous model for our hBN/water/graphite system to extract the structural and, therefore, the density data for the monolayer confined water phase presented in the paper (https://doi.org/10.1063/5.0239693).

In terms of density, it is known from the earlier literature that water develops a non-uniform structure and density in the vicinity of interfaces. The higher packing of water at solid interfaces is then defined by the excess properties of the system. Larger densities for ultra-confined and even monolayer water have been previously predicted by several studies in the earlier literature (please kindly refer to the references 1 to 6 listed below).

A more elaborated description with a complementary HB analysis developed from our simulation is presented below.

A glance over the structure: As extensively reported, water adopts a non-uniform structure and density in the vicinity of interfaces and under confinement. For the hydrophobic case - which is what our system mostly comes close to - the structure and packing of water over or around the surface, strongly depends on both the geometry/curvature and the volumes in question (https://www.nature.com/articles/nature04162). In our systems, the atomically flat surfaces forming the channels are then able to accommodate a much larger population defined by the excess properties of the top and bottom interfaces. In the monolayer regime, these rather packed water layers then overlap, and confinement effects take over interfacial contributions, as suggested by other recent studies on similar systems (https://doi.org/10.1063/5.0239693, https://doi.org/10.1038/s41586-020-2978-1).

Moreover, the physical sub-nanometre constraint prevents the formation of the tetrahedral HB structure as it is pushed down into the planar monolayer configuration. In such scenario, the geometric packing prevents that all molecules can be H-bonded thus leading to a rather **disrupted/frustrated network with fewer HB per water molecule** in average as well as un-bonded water. A better illustration of such structure is presented by the simulation snapshots presented in the updated Fig. 3(h,i) in the main text, depicting the proposed monolayer water phase confined between hBN and graphite in our channels. Such structure, where the vibrational contributions from both dimers and trimers would also emerge at lower frequencies, is consistent with the spectral features experimentally measured and showing a rather broader peak.

To further elucidate the structural moieties forming the monolayer water phase, we performed a detailed statistical analysis of the HB D-A pairs where we correlate the angular distribution with their length (O-O distance). The data is now presented in updated Fig. 3 in the main text. We frame the

correlation map for the monolayer phase directly compared with the bulk counterpart. In both cases, the hottest regions concentrate below 30 degrees angle and around 2.7 Å in length, which are well known as the most stable configuration at room temperature. However, the histogram is clearly re-distributed for the monolayer case, with additional populations emerging at much larger angles, as indicated by the spread wing in the projected plots in panel "f" (Fig. 3f).

Such distortion is not surprising since the physical sub-nanometre constraint prevents the formation of the tetrahedral HB structure as it is pushed down into the planar configuration. In this scenario, the ideal HB angle is then suppressed for a considerable population of water molecules as the pairs stabilise rotated from their ideal angle, thus leading to a frustrated network with fewer HB per water molecule and accommodated among un-bonded water sites. Such mosaic-like structure is presented in the snapshots in Fig. 3h. Interestingly, as this disrupted network comprises a large variety of sites having from none up to 3 HBs, a rather broad spectral response such as those observed in our measurements are expected since the -OH stretching frequencies vary slightly for each HB state.

Supporting references:

1. https://doi.org/10.1103/PhysRevA.44.3732
2. https://doi.org/10.1038/s41586-020-2978-1
3. https://doi.org/10.1021/acs.langmuir.6b02024
4. https://doi.org/10.1021/ja074418
5. https://doi.org/10.1021/la901314b
6. https://doi.org/10.1063/5.0239693

The discussions of all these aspects needs to be extended significantly, also what we learn from the DFT.

We appreciate the reviewer's suggestion and have added the extended discussion based on the points described above.

Minor comments:

I don't understand the aspect of "sub-diffractional" in the title. I don't think it is relevant for the message of the paper

We thank the reviewer for this observation. The term **sub-diffractional** here was used based on the fact that our heterostructure is capable of accommodating resonant wave-guiding modes down to nanometre scales because of the well-known properties of hBN (https://www.nature.com/articles/ncomms6221), and which is far below the diffraction limit of the wavelengths used in our experiments. A second and not less important reason is that, even if only for the normal component, one would not expect to acquire direct observables at the sub-nanometre scale in view of the diffraction limit for the wavelengths of our excitation beam (1 – 10 µm).

Therefore, the authors believe that the capability of directly assessing the vibrational observables far below the diffraction limit with such enhancement secures one of the key experimental novelties of our work, which goes beyond conventional IR micro-spectroscopy.

Page 6, “see AFM topography in Fig 1 b,c)” should probably be Fig. 1d

We thank the reviewer for noticing it. It has now been amended.

In Fig. 1c, the labelling “Bulk” and “Channels” is offset relative to the lines they should describe

We appreciate the reviewer’s kind observation. The figure has been now updated accordingly.

Page 6, “In Fig. 1f, we confirm..” should be Fig. 1g

We thank the reviewer for spotting that out. It has now been corrected.

Page 7: I thought the sentence “As the rotational quanta of energy are in the order of only a few cm-1 (~10-4 eV, primarily in the microwave-THz region of the spectrum), they rather provide valuable insights into rotational modes but offer limited information about the intermolecular forces for small molecules such as water (20). “ was pretty meaningless. Nobody in the community would be surprised to look at the OH stretch vibrations, plus, rotations are not visible anyways in solutions.

We appreciate the reviewer honest observation and agree that this sentence does not add much to the big-picture of the paper. It has now been removed.

Page 8: The sentence “As shown in the representative spectrum from the monolayer case in Fig 1e, the centroid blue-shifts when under confinement.” is the very result of the paper. But it takes 2 pages after introducing the corresponding results in Fig 1 with in part not very relevant discussion (e.g.my previous point) until the authors come to that point. I would recommend restructuring the paper a little bit not to loose readers.

That is a fair observation, and we thank the reviewer for the kind feedback. Indeed, we agree that a restructuring will improve the flow of the paper. This section of the main text has been properly updated.

Page 10: I don’t understand the sentence: “Although HB does not directly influence the band shifts, it affects the lifetime of the intramolecular vibrational modes”. The very point of the paper is that the HB does affect the band shift, the experiments performed don’t measure lifetimes (it is linear spectroscopy), and the lifetime has no effect of the band shift.

We do appreciate the reviewer’s observation. Indeed, we agree that this sentence has been miswritten in the manuscript and could rather lead to an interpretation that is inconsistent with our findings. The initial intent was to build the correlation between intra- and intermolecular nature of our observables. In such frame, one could primarily relate the band-shifts as a consequence of the vibrational changes that water molecules undergo at different states, and in terms of the intramolecular/covalent potentials. Those would result in either stiffer or loser -O-H covalent bond which would thus define the magnitude of the dipole moment and, consequently, the intermolecular force strength (also explored in a later section of the paper with support from our simulation analysis).

Nonetheless, after revisiting the statement, we do agree that such intramolecular perspective does not add to the paragraph in question, which rather evolves describing the system from a HB/intermolecular perspective. Thus the sentence has been removed.

Page 10: Disentangling the IR spectrum of water in the way described in the sentence “ 3140 cm-1,

linked to strongly bound or ice-like water; 3220 cm-1 from tetrahedral water; 3400 cm-1 from tetramers; 3540 cm- 1 from trimers and dimers; and 3600 cm-1 from vapour-like or “free water” is highly controversial, to say the least. Certainly, I would not trust a multi-Gaussian fit to disentangle those species. In fact the authors don’t do such a fit, so why introducing that (in my opinion) very problematic assignment.

We appreciate the reviewer’s word of advice on the sub-mode analysis. In fact, this matter is the core of our follow-up study, which is being finalised and where we analyse the evolution of the stretching mode(s) in the 2-dimensional water in our systems across a wide range of temperatures to then evaluate validity of such structural assignments from the sub-modes, as well as, the entropy evolution at lower dimensions. We do agree with the reviewer that the deconvolution of the stretching mode is still a debated topic in the water research community, especially when those multi-Gaussian fittings are performed with excessive number of free parameters. However, although the studies used as reference for the statement use distinct deconvolution approaches, they all corroborate with the higher frequencies reflecting the reduction of donor-acceptors pairs, which well-aligns with our simulation in a more generic perspective.

We have now added a sentence stating about the different line of thoughts and debates concerning the deconvolution of the IR spectrum of water as a notice for the readers.

Reviewer #2 (Remarks to the Author):

This is a very interesting manuscript that opens a new avenue to experimentally probe confined water down to a single monolayer, where the properties of the H-bond network are substantially altered compared to bulk and interfacial water. The approach is sound and original, and the manuscript deals with an urgent topic of broad interest for interfacial science. In the future, similar studies could be used to learn about how the properties of the confining matrix, e.g., its topology and morphology, can be exploited to template different water H-bond networks. Therefore, I will be happy to support publication after the following remarks are addressed:

We do appreciate the reviewer's positive feedback on our study, both highlighting its originality and impact in the water-research community as a support for future works.

We also thank the reviewer's valuable remarks, which are addressed below.

- It would be nice if the authors could provide more information on what they define as an anomalous phase of water. The decomposition into local water structure is interesting but not enough to get a comprehensive picture: how is the water H-bond network organized, especially for the single monolayer? Which kind of H-bonded pattern do the water molecules form? How does it adapt to the topology and morphology of the surface? Are the free OH groups only oriented out-of-plane (as usually observed as solid-liquid interfaces, e.g., with SFG) or also within the plane due to the high degree of confinement that frustrates the H-bond network? The DFT calculations and MD simulations could be used to provide much more information here, which I think will benefit the broad impact of the manuscript. Figure 3 already provides some of this information, and I would be interested to read more on that.

The authors agree with the reviewer's recommendation for a more elaborate description of hydrogen bonding network forming the water phase suggested by both our observations and simulations in the monolayer regime. Thus, we have performed additional analyses benefiting from our DFT-MD simulation and the discussion is now expanded in the main text. Moreover, Figure 3 has been updated not only with the new simulation data on water confined in hBN/graphite channels, which better describe our system, but also with a deeper statistical analysis of the HB network pattern in terms of the structural moieties forming the monolayer water phase. We correlate the HB angular distribution with their length (O-O distance) and analyse the evolution of common order parameters that show a clear distinction between interfacial and pure confinement contributions. The key-points are outlined below based on the reviewer's request:

To start the description, it is worth to first bring a few remarks regarding the terminology used in the earlier version of the manuscript, still from a local structure perspective. Interestingly, when framing the paper, the authors had mindful discussions regarding the terminology to better describe the water state from a hydrogen-bonding perspective. The term "weaker hydrogen-bonds" has been simply chosen as one could primarily infer it from our observables, i.e. the centroid blue shifts. However, we do agree that the matter is more complex from a local-structure point of view, for which the term "fewer hydrogen-bonds" (per water molecule) would better fit to describe it for the monolayer regime. The DFT snapshots in the updated Fig. 3(h,i) in the main text frames such state in which a disruptive network (represented by the blue-dashed links) emerges as a consequence of the confinement. Non-hydrogen-bonded water would thus form a fraction of such frustrated network. From our simulations, we propose that the average number of hydrogen-bond per water molecule decreases significantly in

the monolayer case (2.56, Fig. S7d) when compared with the tetrahedral arrangement well-known for the bulk (3.66, Fig. S7f).

A structural description of the hydrogen-bonding pattern evolution from bulk to monolayer:

To further elucidate the structural moieties forming the monolayer water phase, we used a deeper statistical analysis of the HB D-A pairs where we relate the angular distribution with their length (O-O distance), where we frame the correlation map for the monolayer phase directly compared with the bulk counterpart. The data is now included in the updated Fig. 3(d-f). In both cases, the hottest regions concentrate below 30 degrees angle and around 2.7 Å in length, which are well known as the most stable configuration at room temperature. However, the histogram is clearly re-distributed for the monolayer case, with additional populations emerging at much larger angles, as shown by the spread wing in the projected plots in panel "e".

This is not surprising since the physical sub-nanometre constraint prevents the formation of the tetrahedral HB structure as it is pushed down into the planar monolayer configuration. In such scenario, the geometric packing prevents that all molecules can be H-bonded thus leading to such **disrupted/frustrated network with fewer HB per water molecule** in average as well as un-bonded water. Such structure, where the vibrational contributions from both dimers and trimers would also emerge at lower frequencies, is consistent with the spectral features experimentally measured and showing a rather broader peak.

Distinguishing the hydrogen-bonding structure among bulk, interfacial, and monolayer confined water:

To gather further insights into how the interface plays a role in the HB structure and whether **water adapts to the topology or morphology of the surface**, we take a closer look into the ordering of the water phases emerging in both pure interfacial and confinement regimes. As a link to the reviewer's next comment, we analyse these correlations in terms of common order parameters with respect to the tetrahedral undercoordination given by the parameter q. In brief, q varies between 0 and 1, where q=1 reflects a perfectly tetrahedral coordination, and q=0 the total absence of order as expected for the extreme un-bonded case. A full description of the method is presented as response to the upcoming comment.

Interestingly, we find significant differences between interfacial and nanoconfined water. In Fig. R1 we compare bulk and interfacial water at the upmost layer on the graphene and hBN open surfaces, against the monolayer water confined between both. For the purely interfacial regimes, we find that the water structure on either graphene or hBN open surfaces are practically indistinguishable. In contrast, the tetrahedral order distribution dramatically changes when these two surfaces are brought together to accommodate the monolayer, splitting into two distinct levels of ordering almost equally present, which we associate with the frustrated HB network described above. Moreover, as depicted in panel "b", no distinction between the order parameter distribution for asymmetric hBN/Graphite channels and symmetric graphite/graphite confinement are observed. Such clear deviation also reinforces our reasoning behind pure confinement effects taking place at the extreme monolayer regime.

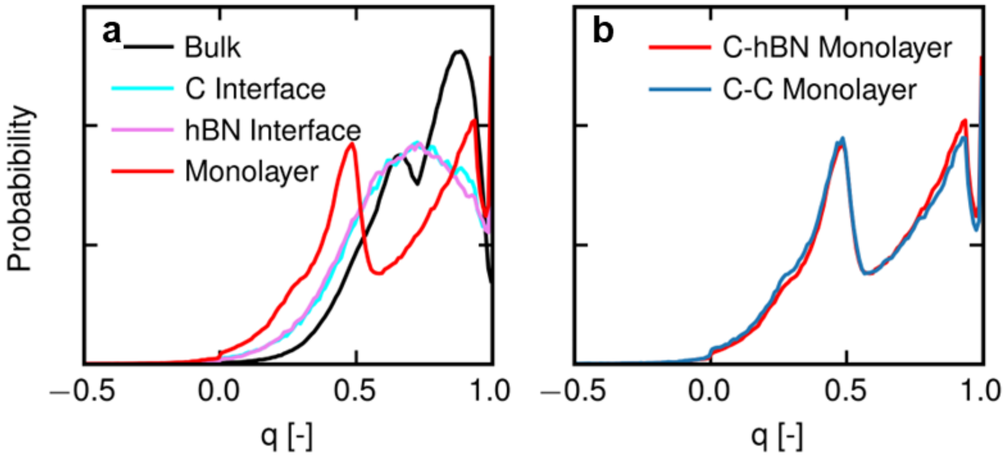

Figure R1: Interface *vs* confinement ordering in monolayer water. (a) Probability density distribution of the order parameter q for bulk (black) and interfacial water arranged on both graphene (cyan) and hBN (pink) surfaces compared with that of monolayer water confined between graphite and hBN. (b) Probability density distribution of the order parameter q for both hBN/Graphite and graphite/graphite confinement.

Therefore, our analysis suggests that water **does not** adapt to the topology or morphology of the surfaces used in our system. This could also explain the compelling spectral similarities for water confined in both systems, graphene-graphene and water in hBN-graphene channels that is now discussed in the manuscript.

The orientation of un-bonded water: from interface to pure 2-dimensional confinement.

The authors thank the reviewer for suggesting a more elaborate view on the free -OH behaviour as the interfacial components swaps into pure confinement effects at the monolayer regime. As these moieties form a significant part of the 2-dimensional HB network presented in our work, we do agree that it can benefit the broader impact of our work.

As the reviewer pointed out, and from several SFG studies, free -OH groups (that is OH groups that are not donating a hydrogen bond) at interfaces are known to be oriented out-of-plane. In the monolayer regime, however, since every -OH group oriented out-of-plane is inherently free due to the confinement constraints, we believe that a **free in-plane population is thus the best descriptor for any unusual structuring**. To dwell into this question, we have used a statistical framework to analyse the orientation of the OH bonds of the overall monolayer water population, which we correlate with the hydrogen-bonding state in the network. Therefore, we analyse the probability of an OH group to be hydrogen-bonded as a function of its orientation angle with respect to the channel surface (namely, 90 degrees for in-plane water and 0 or 180 degrees for out-of-plane-oriented molecules). The results are displayed below and details on the calculation will be added to the supporting information of the manuscript.

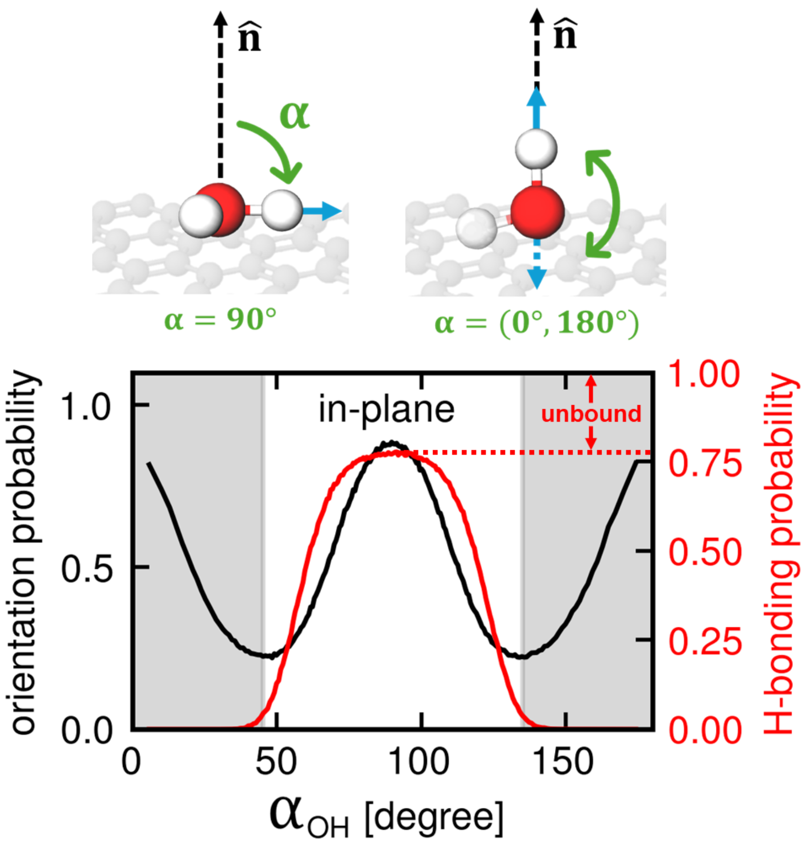

Figure R2: Orientation distribution of water molecules in the monolayer phase. The schematics in the upper panel represent the orientation condition (α) with respect to the vector $\hat{n}$, normal to the channel surface. The analysis obeys the condition that all the water molecules oriented out-of-plane (**α**=[$\mathbf{0°}$, $\mathbf{180°}$]) do not make part of the H-bonding network thus considered free. The black curve indicates the overall orientation distribution shown nearly equal probabilities for in- and out-of-plane OH group, whereas the red curve gives the probability density that the respective OH takes part in a hydrogen bond. For the latter, the complement indicated by the dashed red line reveals that nearly 25% of the in-plane OH groups are rather free (disruptive sites), which contrasts what is known for the bulk or near simple open interfaces.

Interestingly, for the overall water molecules population, in- and out-of-plane OH groups are distributed nearly equally in the monolayer phase. Such arrangement already differs from the bulk, where all orientations are uniformly present. As noted before, all out-of-plane OH groups (< 45 degrees and > 135 degrees) are also free. For the in-plane populations, while the majority of groups is hydrogen-bonded, still a significant proportion of nearly 25% remains free. This stands in strong contrasts to what is known for bulk water, where around 95% of all OH groups are hydrogen bonded, and to interfacial water, where free OH groups are predominantly outwards/perpendicularly oriented.

Therefore, we propose that such structural feature supports the concept that simply interfacial effects do not dictate the confined monolayer water structure, as one could expect the entire free-OH population to be oriented out-of-plane in such case. Instead, confinement effects not only dominate but also define such unusually disrupted HB structure.

- Also, some information on the water dynamics, as well as the evaluation of the common order parameters for water, may be insightful to understand to what extent this can be defined as an

anomalous phase

We appreciate the reviewer's suggestion to evaluate the structure of the confined water phase *via* the extent of its ordering. This will certainly help the readers to better frame our findings.

We have calculated the orientational tetrahedral order parameter popularised by Errington & Debenedetti (https://doi.org/10.1038/35053024), which has been recently adapted by Wang *et al* (https://arxiv.org/pdf/2504.05593) to better characterise the structure of interfacial water in terms of the angles between neighbouring water molecules. The local structure of the hydrogen bonding network is quantified by the orientational tetrahedral order parameter q, which is based on the angles ψ_{jk} that are formed between the vectors connecting oxygen atoms among the nearest neighbouring water molecules j and k.

We then compute the order parameter to take the undercoordination of interfacial water molecules into account, according to Wang *et al*:

$$q = \begin{cases} 1 - c_N \sum_{j=1}^{N-1} \sum_{k=j+1}^{N} \left(\cos\psi_{jk} + \frac{1}{3}\right)^2 , & \text{if } N \geq 2 , \\ 0 & \text{if } N < 2 , \end{cases}$$

With $c_N = 9/(2N(N-1))$, where N is the number of neighbours of a water molecule found below a cutoff radius of $r_{OO} < r_c = 3.19$ Å. This cutoff radius results into an average of 4 nearest neighbouring molecules in our bulk water simulation. In short, if it has a value of 1, the hydrogen bond structure would be perfectly tetrahedral. If it takes a value of 0, it has no order at all corresponding to an ideal gas. The figure below summarises the results and structural insights based on the reviewer's recommendation.

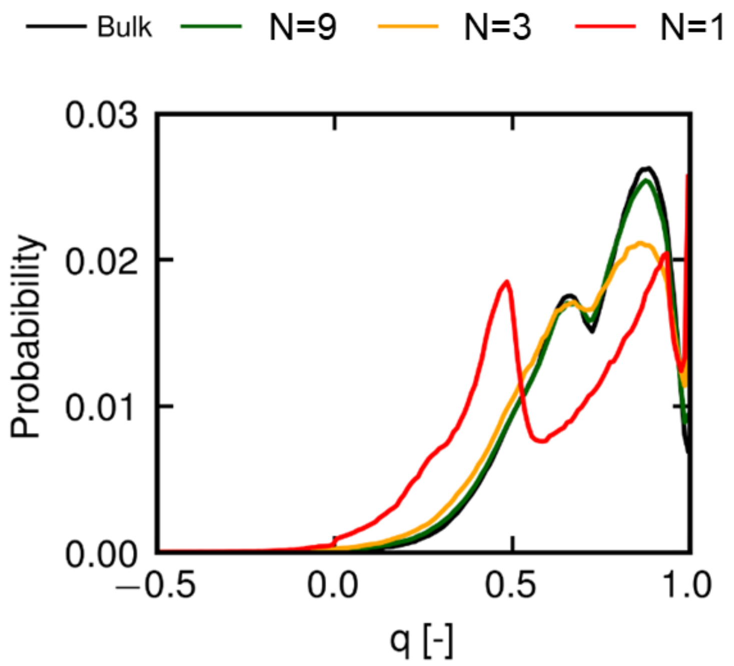

Figure R3: Order distribution of water at various levels of confinement between hBN and graphene. The figure depicts the order parameter analysis for various channel heights in comparison with bulk showing a strong distinction for the monolayer phase.

Figure R3 evaluates the distribution function of this order parameter q in the entire system as a relative to the graphene sheet separation. As expected, our model shows that for thicker 2-D capillaries, bulk-like water dominates, showing the order parameter distribution comparable with bulk used as reference. Therefore, we can define three structurally distinct populations of hydrogen-bond network: bulk-like within thicker channels, interfacial water along the first hydration layer of the surface, and monolayer water as its own kind. Additionally, as described in the response to the previous comment, the water structure at graphene and hBN interfaces alone are nearly identical, whereas the distribution function of q for the monolayer is notably different, exhibiting two distinct well separated peaks. This supports our hypothesis that the structure of water in the monolayer is fundamentally different not only from bulk water, but also from interfacial water.

A new section on the water ordering analysis has been added to the updated supporting information. The full set results are presented in Fig. S8.

- I would also like to read more about the transition in the spectroscopic signature and water structure when going from 10 to 1 water layer. A deeper comparison with previous observations for confined water in the literature could be beneficial. The picture I have in mind from previous studies is that, in terms of the structure and spectroscopic response (as measured in SFG spectra), the topmost water monolayer in contact with the surface doesn't change dramatically upon increasing the degree of confinement down to 3 water layers (e.g., ref.https://www.nature.com/articles/s41467-025-62625-w [nature.com]). Please elaborate.

Indeed, that is a valid point, and we thank the reviewer for suggesting a more elaborate discussion on this view benefiting from the recent literature support. Firstly, we recognise its relevance not only because it corroborates with previous studies but also fills the still-standing gap on the experimental side for the true monolayer water limit. Overall, both our experimental observations and theoretical analyses points to the existence of three distinct hydrogen bonding environments: bulk-like, interfacial, and purely confined (monolayer case).

The recent work nicely referenced by the reviewer is, in fact, very consistent with our findings, especially regarding the transition in our measured blue shift amplitudes for channel thicknesses above 3 graphene layers. The deeper hydrogen-bonding analysis that we now add into the study indeed suggests that the interfacial water layers on both graphite and hBN surfaces remain almost unchanged until confinement effects become prominent as the channel narrows down even further. Interestingly, and also still in terms of SFG spectra, such transitions in the spectroscopic response down to a few water layers have been predicted even earlier by B. Das, S. Ruiz-Barragan & D. Marx (2023) (https://doi.org/10.1021/acs.jpclett.2c03409). Moreover, and as also mentioned in our work, they also emphasised the challenges of measuring reliable SFG signal for symmetrical monolayer confinement (two interfaces alike). Finally, from a structural perspective, the very recent study by Advincula, C. Schran, and A. Michaelides (2026) (https://doi.org/10.1039/D5FD00165J), where they used machine learning molecular dynamics with first-principle accuracy, also proposes that for three or more water layers confined between graphene surfaces, the interfacial water structure is effectively

indistinguishable from that of an open system, with density, hydrogen-bonding structure, and orientational ordering retaining interfacial character.

Therefore, upon the reviewer's suggestion, we now added a new section to our discussion where we correlate our findings with those in the most recent literature from both experimental/spectral and theoretical/structural perspective, as follows:

"To enclose the link with our experiments, the transition in the blue shift amplitudes agrees well with predictions made along the last decades (69-71) and SFG experiments (10). Although the latter presents the measured quantities until degrees of confinement still slightly above the monolayer limit, the three-layers threshold also stands out as a cross-over, where the spectral signal starts varying as consequence of a restructuring HB network imposed by Ångström-scale confinement. In fact, a theoretical study performed earlier by Das et al (71) has been recently further explored by Advincula et al (70) then focusing on water's structural evolution using novel machine learning-assisted MD simulations. Interestingly, their findings also suggest that on graphene surfaces at lower degrees of confinement, the orientational ordering of water is rather similar to that found in open graphene-water interfaces, dominated by the bulk with most of the free -OH populations oriented out-of-plane at the uppermost layer. Here, however, we show that the same behaviour emerges at the water-hBN interfaces thus justifying the nearly indistinguishable spectral features. As we confine water down to bi- and monolayer regime, strong confinement effects reshape the HB network so that the interface-like ordering is supressed thus giving place to such frustrated network, explaining the strong spectral shifts observed in our measurements. Surprisingly, at the extreme monolayer case, our analysis suggests that a considerable population of nearly 25% percent of in-plane bonds are also free, contrasting what is well-known for simple interfacial systems or bulk."

Reviewer #3 (Remarks to the Author):

Referee report on manuscript NCOMMS-25-58925-T

This manuscript reports a direct experimental observation of vibrational absorption in a monolayer-confined water, which is a very relevant scientific feature. The authors employ narrow slit capillaries built from van der Waals heterostructures (graphite + hexagonal Boron Nitride) and synchrotron infrared microspectroscopy to detect OH-stretching signatures at single-layer resolution. The measurements reveal significant blue shifts consistent with a weakened hydrogen-bond network, supported by DFT-MD simulations. The data provided in this manuscript suggest that confined water forms a dense but weakly hydrogen-bonded phase, a structural regime distinct from both bulk liquid and vapour. In my opinion, this is not a truly novel statement, since previous works have observed different water phases though peculiar hydrogen-bond rearrangements. Lacking references (see below) can enlighten the readers about this point.

On novelty and impact, this work provides direct and unprecedented experimental access to the vibrational structure of monolayer water, a topic of broad interest in nanofluidics, interfacial science, and condensed matter physics. Further, DFT-MD simulations corroborate experimental findings overall, particularly HB weakening, density increase, and anisotropy in vibrational response. On methodology, the device design combining graphite reflectivity and hBN waveguiding to amplify weak IR absorption is pioneering and it can be employed beyond this work. The use of synchrotron IR microspectroscopy avoids common heating artefacts. Finally, the authors report clear and reproducible spectral shifts with strong control measurements (empty vs filled channels, bulk vs confined).

We appreciate the reviewer's constructive feedback on our work and its relevance to the field of nanofluidics and beyond.

Before elaborating on the aspects to improve that were nicely recommended by the reviewer, we would like to clarify the point risen regarding one of core statements of our work, which is the weaker hydrogen bonding in the confined phases.

When framing the paper, the authors had mindful discussions regarding the terminology to better describe the water state from a hydrogen-bonding perspective. The term "weaker hydrogen-bonds" has been simply chosen as one could primarily infer it from our observables, i.e. the centroid blue shifts. However, we do agree that the matter is more complex from a local-structure point of view, for which the term "fewer hydrogen-bonds" (per water molecule) would better fit to describe it. The DFT snapshots in Fig. 3b in the main text frames such state in which a disruptive network (represented by the blue-dashed links) emerges as a consequence of the confinement. Non-hydrogen-bonded water would thus form a fraction of such frustrated network. From our simulations, we propose that the average number of hydrogen-bond per water molecule decreases significantly in the monolayer case (2.56, Fig. S6d) when compared with the tetrahedral arrangement well-known for the bulk (3.66, Fig. S6f).

The same picture can be formed from a geometric analysis. We are now complementing the supplementary Fig. 3 of the main text with an additional data on the hydrogen-bonding angle distribution. Whereas the 30 degrees geometric criterion prevails for the bulk and 'bulk-like' case in thicker channels, a wing indicating wider hydrogen-bonding angles emerges towards the limiting monolayer case.

As for the proposed aspects to be improved, they are separately elaborated below:

Aspects to improve:

The study relies on centroid shifts of the -OH band. A more detailed sub-band decomposition (even constrained by simulations) could yield deeper insights into HB motifs.

We are glad that the reviewer suggests a deeper analysis of the sub-modes of the stretching vibrations and agree that deeper insights can be built from it. Interestingly, this is the backbone of our upcoming work that is currently being finished and where we use synchrotron IR to analyse the structure of the HB network through the -OH stretching sub-modes over a broad range of temperatures. Besides the structural phase evolution down to the monolayer regime, we aim to gather insights into the entropic contributions defining such phases.

We are aware that such deconvolution into Gaussian sub-bands still sparks debate across the community, and believe that problems mostly (to not say only) emerge based on the fitting protocol adopted by different authors. Therefore, in our upcoming work, we will also explore the validity or accuracy of such models by having a much deeper perspective on how these parameters evolve along a wide range of temperatures since it has direct effects on the width of the overall band, HB ordering across the low temperature phases, and sub-mode positions. As of now, and upon the reviewer's kind observation, the authors discussed and decided to remove the sentence in the main text that brings up this deconvolution analysis. It may not be relevant to this study.

On the comparison to simulations, my remark is that, whereas DFT-MD simulations model graphene confinement, the experiments use graphite + hBN. The possible differences and limitations of this approximation deserve fuller discussion. Direct comparison is not trivial.

That is a valid point, and we do agree with the reviewer kind observation that approximations on the modelled channel structure is not trivial and could lead to inconsistencies with our observations. Such approximation was used by initially assuming that both graphene and hBN would have similar contribution on the confined water phases due to their comparable hydrophobic nature and identical topology (i.e., both atomically flat). However, the authors reconsidered and now agree that to better reflect our experiment, an adapted the model accounting for both graphite and hBN would be necessary to completely rule out any differences or limitations imposed by different channel structures. The simulation results and respective discussions have been updated in both main text and supporting information, with complementary statistical analyses on the hydrogen-bonding structure and the levels of tetrahedral ordering (in the supporting information). Please, kindly refer to the revisited Figure 3 in the main text, we introduce the simulation results along with a supporting statistical analysis on the hydrogen-bonding structure. Briefly, we have noticed that both systems work almost identically leading to the same extent of blue-shifts observed in our measurements.

The study focuses exclusively on graphite–hBN confinement. A discussion of how results may (or may not) extend to other hydrophobic or hydrophilic environments would strengthen the impact. Carbon nanotubes would be an excellent candidate.

We thank the reviewer for suggesting a comparison with other interfaces or systems to broaden the significance of our findings. In fact, in the discussion part titled “Spectral signature of water under extreme confinement” we used a whole paragraph to trace a parallel with very recent findings on water confined in carbon nanotubes. The section is highlighted below. As an one-dimensional analogue, these systems become extremely relevant and not only corroborate with our findings regarding the confinement effects in the vibrational modes, but also complements the understanding of water under extreme confinement. Moreover, their results were obtained using a totally different methodology, where the vibrational modes of the confined water phases are assessed *via* the energy loss of an incident electron beam.

“Similar effects have also been measured for water confined in carbon nanotubes (CNTs) where the stretching mode was measured for confining diameters down to 1.4 nm. These measurements were obtained using a completely different technique—vibrational energy loss spectroscopy (vEELS)—and further supported by DFT-MD simulations. In this one-dimensional analogue, the authors also found that bulk effects vanish for CNT with 1.4 nm in diameter and a vapour-like water phase emerges. These findings help refine our understanding of how water molecules behave in hydrophobic spaces with dimensions comparable to the hydrogen-bond (HB) length—a highly significant yet elusive concept with implications for many systems.”

This section was elaborated in reference to the work done by Xu and co-authors: X. Xu et al., Hydrogen bonding in water under extreme confinement unveiled by nanoscale vibrational spectroscopy and simulations [arXiv preprint arXiv:2402.17989, (2024)].

Additionally, as for hydrophilic environments, which template a totally different structure of both interfacial and confined water, with the support of several references, we have briefly discussed in the third paragraph of the introduction section how strong correlations between water and the host structure has direct effects on the hydrogen-bonding network, thus masking the actual water-water interactions.

“Developing a suitable host structure to study the properties of pristine water single layers to unravel the elusive properties of HB network has long been a challenge. Crystalline laminate materials such as graphene oxide (20), clays, gypsum and other crystalline systems potential candidates and were extensively used to fulfil such role in X-Ray and neutron crystallography as well as vibrational spectroscopy (11, 21-26). However, strong correlations between water and these hydrophilic environments outweighs the water-water correlations framing the bare HBs, which thus remained obscure. The discovery of 2D materials and their portfolio of features such as being atomically flat and primarily hydrophobic holds good promises in this respect.”

These sections have now been improved with the respective references to previous studies.

The conclusion of a threefold density increase in monolayer water is based on indirect reasoning. Without direct structural probes (e.g., X-ray or neutron scattering), this remains somewhat speculative. Verify or remove this conclusion.

We thank the reviewer for such valuable observation and do agree that direct structural probes would be essential to validate such density profiles. However, the scales of both our channels and the device as a whole, rather offer a cross-section that is much shorter than the minimal required for scattering-based techniques such as X-Ray and neutron scattering. For the former, the limitation arises from the lower scattering factor of oxygen whereas H is undetectable. Thus, a single to a few planes of water disposed within the heterostructure would be undetectable for most conventional hard X-Ray diffractometers even at the synchrotron nano-probe regime available at Diamond Light Source. Moreover, at such high collimation of the coherent synchrotron beam, sample damage would be a problem hard to mitigate. As for neutron scattering, the problem strictly lies on the very small scattering cross-section of our samples. Speaking of water, whereas a common neutron scattering experiment requires at least a few hundreds of micro-litres, each of our channels are in fact within the zepto-litre scale, i.e. 15 orders of magnitude smaller than the minimum required. Even with the enhanced neutron scattering length of hydrogen, our scales would prevent any reliable data from such probes.

The simulation-based insights into the density under confinement have been widely used for other systems at lower dimensions, including carbon nanotubes and nanochannels like ours. One can refer, for example, to the study of capillary condensation at sub-nanometre scales, ion transport along these channels, and others. Although such density increases under nanoconfinement seem sound from the perspective of the well-known interface and excess properties, we do agree with the reviewer that introducing these results on a quantitative basis in one of the main figures might not be the best way, as we lack direct experimental measurements to validate or falsify it. Therefore, we have now changed the statement of a three-fold density increase previously used.

On references: This manuscript lacks some context on previous works related to the confinement of water in reduced dimensions and its behaviour at interfaces. Concerning the experimental side, I will mention works published by Skinner at al. (Acc. Chem. Res. 2012, 45, 1, 93–100, https://doi.org/10.1021/ar200122a [doi.org] ; Nature 474, 192–195 (2011). https://doi.org/10.1038/nature10173 [doi.org] or PNAS 104 (36) 14215-14220 (2007). https://doi.org/10.1073/pnas.0701482104 [doi.org]) and works form Bakker et al. (Chem. Rev. 2010, 110, 3, 1498–1517, https://doi.org/10.1021/cr9001879; [doi.org] PNAS 102 (9) 3202-3207 (2005) https://doi.org/10.1073/pnas.0404916102 [doi.org]) among others. Form theoretical-computer simulations, works indicating the alteration of water properties under strict confinement, such as inside carbon nanotubes, for instance, are also worth to be mentioned: Acc. Chem. Res. 2017, 50, 9, 2139–2146. https://doi.org/10.1021/acs.accounts.6b00617 [doi.org] ; Phys. Rev. B 63, 165430 (2001). https://doi.org/10.1103/PhysRevB.63.165430 [doi.org] ; Sci Rep 15, 28144 (2025). https://doi.org/10.1038/s41598-025-14266-8 [doi.org] ; J. Phys. Chem. C 122, 6684–6690 (2018). https://doi.org/10.1021/acs.jpcc.8b00112 [doi.org] ; J. Am. Chem. Soc. 2006, 128, 37, 12090–12097. https://doi.org/10.1021/ja057856u [doi.org] , etc. All these references (and many others) are relevant to focus the reader in the appropriate context, what is important to emphasize the novelty and relevance of the present work. This drawback is one of the issues that need to be addressed.
Improve figure clarity and streamline technical sections for accessibility.

We appreciate the reviewer's rich set of suggestions for reference and do agree that they will reinforce the context and relevance of our work. As indicated below, all the citations were included along with a more elaborated discussion, especially on the theoretical side where further insights into the hydrogen-bonding structure as well as its ordering were added.

- Acc. Chem. Res. 2012, 45, 1, 93–100, https://doi.org/10.1021/ar200122a [doi.org]: The reference was added to the introduction section when referring to the properties at interfaces of different nature, i.e. hydrophilic and hydrophobic.
  "For decades, vibrational spectroscopy has been an effective tool for probing water's structure and HB environment. In particular, the water molecule stretching modes offer a detailed picture of its HB network, revealing distinct changes compared to bulk water as it is scaled down to smaller number of molecules or at interfaces (11-15). Nonetheless, the limited cross-section in the actual monolayer regime is a major challenge, preventing these subtle structural features from being experimentally resolved, even by state-of-the-art spectroscopy techniques. For example, sum-frequency generation (SFG) spectroscopy recently emerged as a promising approach due to its transient aspect and interface sensitive hallmark (16-19)."

- Nature 474, 192–195 (2011). https://doi.org/10.1038/nature10173 [doi.org]

  "From several SFG studies, free -OH groups (or -OH groups that are not donating a hydrogen bond) at interfaces are well known to be stabilised out-of-plane relative to the surface (62)."

- PNAS 104 (36) 14215-14220 (2007). https://doi.org/10.1073/pnas.0701482104 [doi.org]

  "The overall -OH stretching band is well known as a superposition of multiple normal modes with its frequency/position defined by the number of water molecules participating in the donor-acceptor configuration of the HB network. For bulk water, the combined stretching band centred at ~3400 cm-1 builds as a superposition of different sub-modes (13-15), each one related to a distinct HB environment. Although the exact positions of these sub-modes are still debated (51,52), the centroid of the overall stretching band does depend on their relative intensity."

- Chem. Rev. 2010, 110, 3, 1498–1517, https://doi.org/10.1021/cr9001879; [doi.org]

  "The overall -OH stretching band is well known as a superposition of multiple normal modes with its frequency/position defined by the number of water molecules participating in the donor-acceptor configuration of the HB network (51). For bulk water, the combined stretching band centred at ~3400 cm-1 builds as a superposition of different sub-modes (13-15), each one related to a distinct HB environment."

- Acc. Chem. Res. 2017, 50, 9, 2139–2146. https://doi.org/10.1021/acs.accounts.6b00617 [doi.org]

  "The structure shaped by its hydrogen-bonding (HB) network at lower dimensions, defines water's energy landscape and gives rise to the unusual physical-chemical properties (3), such as ultrafast flow (4-6) and a highly anisotropic dielectric function (7-9)."

- Phys. Rev. B 63, 165430 (2001). https://doi.org/10.1103/PhysRevB.63.165430 [doi.org]

  "To enclose the link with our experiments, the transition in the blue shift amplitudes agrees well with recent predictions (66-68) and SFG experiments (69)."

In summary, this work addresses a long-standing challenge in water science, bridging the gap between theoretical predictions and experimental validation. It represents a significant technical and conceptual advance in the study of confined liquids. All in all, it is a technically impressive and conceptually important contribution that will attract broad interest. I recommend major revision following the lines indicated above, before eventual acceptance.
